# Roles of hormones, calcium and *PmWRKY31* in the defense of *Pinus massoniana* Lamb. against *Dendrolimus punctatus* Walker

**DOI:** 10.48130/FR-2021-0021

**Published:** 2021-12-03

**Authors:** Hu Chen, Ying Hu, Xingxing Liang, Junkang Xie, Huilan Xu, Qunfeng Luo, Zhangqi Yang

**Affiliations:** 1 Guangxi Key Laboratory of Superior Timber Trees Resource Cultivation, Masson Pine Engineering Research Center of the State Forestry Administration, Masson Pine Engineering Research Center of Guangxi, Guangxi Forestry Research Institute, Nanning 530002, PR China; 2 Guangxi University, Nanning 530002, PR China

**Keywords:** WRKY transcription factor, Cakcuyn bubdubg oriteub, Hormonal signals, Plant defense, *Pinus massoniana* L.

## Abstract

*Dendrolimus punctatus* Walker is a major pest affecting *Pinus massoniana* Lamb. forests and can cause serious economic and ecological losses. WRKY transcription factors play important roles in coping with various environmental stresses and plant responses against herbivorous insects. However, the mechanisms underlying the actions of WRKY in the defense responses of *D. punctatus* in *P. massoniana* are still unclear. Our previous study provided evidence that WRKY plays an important role in the insect resistance of *P. massoniana*. In this study, the treatments of exogenous hormones and Ca^2+^ increased the concentrations of endogenous hormones, and terpenoid synthases in *P. massoniana* effectively improving its resistance to *D. punctatus*. After analyzing the WRKY family of *P. massoniana*, *PmWRKY31* was selected and studied. A direct interaction between PmWRKY31 and PmLp8 was observed by yeast double hybridization assay. Gene expression analysis showed that treatments of exogenous hormones and Ca^2+^ induced high *PmWRKY31* expression. The expression pattern of *PmWRKY31* was different under treatment of MeJA compared to those of GA, ABA and SA. These results indicated that *PmWRYK31* and *PmLp8* interacted with each other to promote the expression of terpenoid synthase genes and increase the content of terpenoid volatile substances by regulating the gene expression of hormone signaling pathways, to improve the ability of *P. massoniana* to resist *D. punctatus*, providing theoretical support for the involvement of WRKY transcription factors in enhancement of the insect resistance of *P. massoniana* through their regulation of hormone signaling.

## INTRODUCTION

Herbivorous insects are pests affecting agricultural and forestry production and can result in severe economic and ecological losses. The interaction between plants and insects can activate defense responses in plants^[1]^. This behavior is the first line of defense for plants. It involves the activation of different signal transduction pathways and downstream chain reactions, and the related transcription factors regulate defense gene transcription to synthesize special defense compounds and initiate defense responses^[2]^.

The methyl jasmonate (MeJA), salicylic acid (SA), ethylene (ET), and Ca^2+^ signaling pathways have close relationships and play important roles in plant defense against insects^[3−11]^. Silencing the of MeJA biosynthesis-related genes *OsHI-LOX*, *AOS1*, and *AOS2* significantly reduces the damage to rice plants by brown planthoppers and is regulated by transcription factors such as *OsERF3*, *OsWRKY70*, and *OsWRKY24*^[7−9,12]^. Two plant hormones modulating inducible defenses are SA and jasmonic acid (JA)^[13,14]^. SA plays important roles in inducing insect resistance^[13]^. MeJA and SA can mutually induce defensive gene expression, indicating synergy between the two^[14]^.

The expression of defense genes, accompanied by Ca^2+^ flow and changes in intracellular MeJA and SA in plants subjected to feeding stress^[15]^. Ca^2+^ and Ca^2+^-dependent protein kinase regulation is critical for enhancing the resistance of Arabidopsis to *Spodoptera littoralis*^[16,17]^. In the process of plant resistance, Ca^2+^, SA and MeJA cross-talk and enhance plant resistance through signaling cascades^[18]^.

All plants have their own 'substance' to repel herbivorous insects, and determining the genes that regulate certain substances to 'reject' herbivorous insects from feeding is key^[9]^. Plants can generate many secondary metabolites, especially volatile metabolites such as terpenoids, to avoid harm from herbivorous insects. In the process of plant defense against herbivorous insects, the production of secondary metabolites is regulated by various hormones, such as MeJA, SA, and gibberellin (GA)^[19]^. During terpenoid synthesis, terpenoid synthases (TPSs) directly affect the compound synthesis of terpenoids. Terpenoid compounds become important signaling molecules in plant-environment interactions and are involved in plant defense responses^[20]^. More than 200 TPS genes have been cloned in more than 40 plants^[21]^. In gymnosperms, terpenoid production is induced in roots under stress conditions, e.g., terpenoid compound synthesis increases in *Picea sitchenrsis* after MeJA treatment^[22]^; SA significantly improves plant resistance to insects^[23,24]^.

WRKY transcription factors play important roles in regulating resistance to insects, diseases, and abiotic stress in plants^[25]^. Among the WRKY transcription factors, *WRKY3* and *WRKY6* can regulate the insect resistance of tobacco plants and participate in the MeJA pathway^[26]^. WRKY genes participate in the mechanism of aphid and nematode resistance in tomato and Arabidopsis^[27,28]^. Systematic studies of rice WRKY transcription factors in insect defense have shown that they participate in a variety of hormone metabolic pathways to enhance insect resistance in rice^[29,30]^.

*Pinus massoniana* Lamb. is a very important timber species in China, for more than half of the growing stock of forests in south of China and is also the major resin-producing species in worldwide^[31]^. *D. punctatus*, affecting tree growth and forest ecology, causes serious damage to approximately 140,000 hectares of *P. massoniana* forests every year^[32]^. In our previous study, we sequenced the transcriptomes of insect-resistant strain and insect-sensitive strains of *P. massoniana*. The results revealed that several important transcription factors (TFs), including WRKY and Apetala2/ethylene responsive factor (AP2/ERF), and abietadiene synthase, played significant roles in anti-pest responses^[33]^. Currently, the defense mechanism of *P. massoniana* against *D. punctatus* remains unclear. To understand the defensive signaling pathways of *P. massoniana*, we focused on how WRKY transcription factors play roles in this defense process. This study investigated the mechanisms of the WRKY-based defense system by screening their interacting genes, analyzing changes of hormones, and detecting volatile substances in *P. massoniana*.

## RESULTS

### Mechanisms of WRKY transcription factors in insect resistance

**Figure 1:**
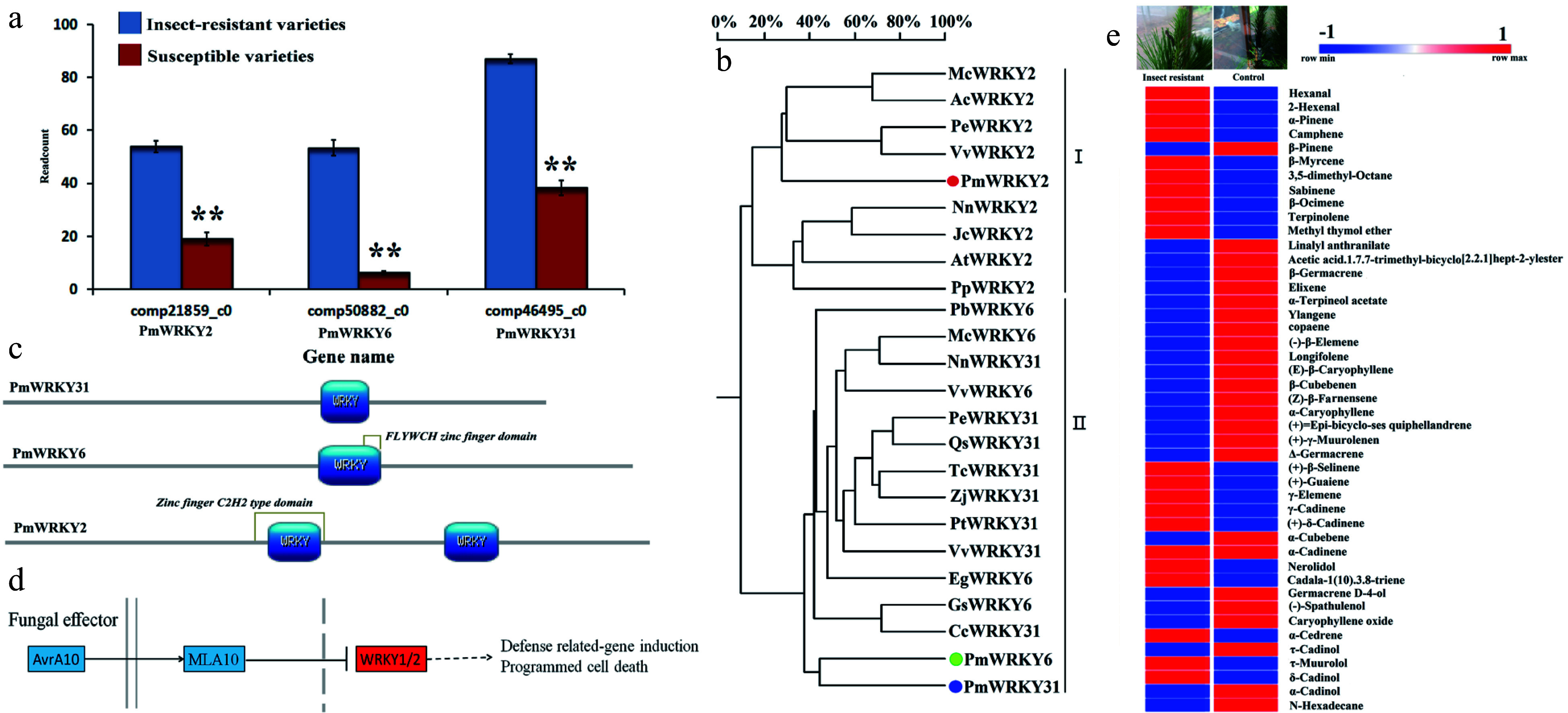
WRKY gene discovery and bioinformatics analysis. (a) Three WRKY transcriptomes obtained from 511 differentially expressed genes in high throughput sequencing analysis of the transcriptome of insect-resistant varieties vs susceptible varieties. The data were read counts of insect-resistant varieties and susceptible varieties, and each sample transcriptome was replicated three times. (b) The analysis of the WRKY polygenetic tree. Pm, *Pinus massoniana* L.; Nn, *Nelumbo nucifera*; Vv, *Vitis vinifera*; Qs, *Quercus suber*; Pt, *Populus trichocarpa*; Zj, *Ziziphus jujube*; Cc, *Cajanus cajan*; Jr, *Juglans regia*; Tc, *Theobroma cacao*; Mc, *Macleaya cordata*; Ac, *Aquilegia coerulea*; Pp, *Physcomitrella patens*; At, *Amborella trichopoda*; Jc, *Jatropha curcas*; Pe, *Populus euphratica*; Gs, *Glycine soja*; Pb, *Pyrus* x *bretschneideri*; Eg, *Elaeis guineensis*. The different WRKYs include NnWRKY31 (XP_010252466.1); VvWRKY31 (XP_002269696.2); QsWRKY31 (XP_023921697.1); PtWRKY31 (XP_002321134.3); ZjWRKY31 (XP_015877768.1); CcWRKY31 (XP_020234210.1); JrWRKY31 (XP_018811738.1); TcWRKY31 (EOX93243.1); NnWRKY2 (XP_010270167.1); PpWRKY2 (XP_024368161.1); AtWRKY2 (XP_006836767.1); VvWRKY2 (CBI39865.3); JcWRKY2 (XP_012070967.1); ZjWRKY6 (XP_015877768.1); QsWRKY6 (XP_023921697.1); GsWRKY6 (KHN36523.1); NnWRKY6 (XP_010252466.1); PbWRKY6 (XP_018502314.1); McWRKY6 (OVA03405.1); EgWRKY6 (XP_010926185.1); PeWRKY6 (XP_011047241.1); VvWRKY6 (XP_002263115.1). (c) NCBI blasts were prediction and SMART and Motif Scan online software were used for functional domain analysis of four genes PmWRLY2, PmWRKY6, PmWRKY31, and PmLp8, which were mapped with PROSITE software (https://prosite.expasy.org/mydomains). Colored sections are the main functional domains of the genes. (d) The annotation and signaling pathways of the WRKY2 gene using the KEGG database. (e) Results for the needle volatile substances of insect resistant varieties and susceptible varieties in May. The results were analyzed by GC and GC-MS (HP6890 gas chromatograph ((Hewlett-Packard Company, USA), GCMS-QP5050A gas chromatography-mass spectrometry (Shimadzu Corporation, Japan)). The sample was repeated three times.

Three *PmWRKY* genes were significantly expressed in insect-resistant *P. massoniana* varieties (Fig. 1a). According to their cDNA sequences obtained from transcriptome data, the three genes were *PmWRKY2*, *PmWRKY6*, and *PmWRKY31*, which encode 667, 575, and 642 amino acids, respectively (Supplemental Table S1). All three genes had the typical WRKY domains of the WRKY family (Fig. 1b & c; Supplemental Table S1). *PmWRKY31* was successfully annotated in the KEGG Orthology (KO) database, indicating that *PmWRKY31* was involved in plant pathogen defense (Fig. 1d). Therefore, we hypothesized that WRKY might be involved in the defense of *P. massoniana* against herbivorous insects.

Seventy-four volatile substances were detected, 45 of which were identified in insect-resistant and non-insect-resistant materials. Among the identified volatile substances, 30 were terpenoids. The *α*-pinene and *β*-pinene contents were increased (Fig. 1e). TPSs and hormones play roles in insect resistance^[29,30,34,35]^. Whether both WRKYs and TPSs participate in the insect resistance of *D. punctatus* and their relationships with hormones and calcium need to be clarified. *PmWRKY31* expression was the highest (Fig.1a); therefore, we analyzed the *PmWRKY31* gene in the following study.

### Screening of PmWRKY31 transcription factor interaction genes

To further investigate the function of PmWRKY31 in the insect resistance of *P. massoniana*, we constructed a cDNA library (Supplemental Fig. S1) and a two-hybrid bait system (Supplemental Fig. S2). The bait strains did not self-activate and had no toxicity (Supplemental Fig. S3a). They were highly expressed in the positive control according to the western blot (Supplemental Fig. S4a). Two colonies (Supplemental Fig. S3b−d) were obtained by two-hybrid screening and four-hybrid screening (Supplemental Fig. S4b). The positive control and negative control showed the expected results (Supplemental Fig. S4c). Sequencing of the PCR amplification product revealed calcium-binding protein (PmLp8) (Supplemental Fig. S4d). PmLp8 contained three EF-hand calcium-binding domains and a secreted protein acidic and rich-in-cysteine Ca^2+^-binding region (Supplemental Fig. S4e).

### Confirmation of the interaction between PmWRKYY31 and PmLp8

**Figure 2:**
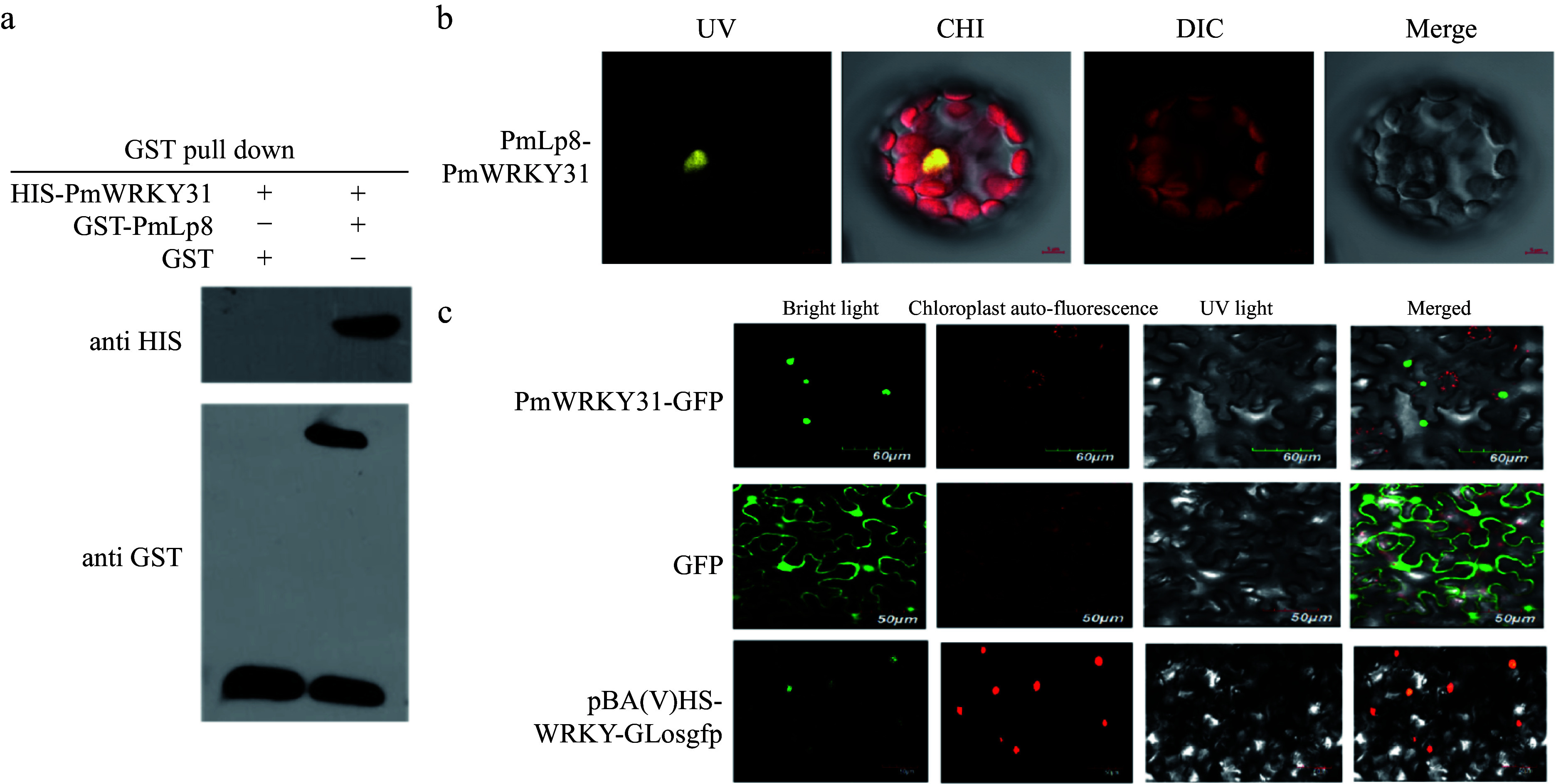
Interaction between PmWRKY31 and PmLp8 *in vitro* and *in vivo*. (a) Pull down experiments of PmWRKY31 and PmLp8 *in vitro*. A GST prokaryotic expression vector for the bait PmLp8 protein and a prokaryotic expression vector of the prey PmWRKY31 protein were constructed. Western blotting was performed after adding loading buffer to GST-PmLp8 and His-PmWRKY31 fusion proteins to verify the normal expression of the fusion proteins. The GST protein and GST-PmLp8 protein with GST resin were incubated with His-PmWRKY31 protein overnight and eluted with reduced glutathione the next day. The following day, elution was performed with reduced glutathione. Western blotting was performed after an appropriate amount of the eluate was treated with loading buffer. (b) BiFC-validated interactions of PmLp8 and PmWRKY31. Constructed of pSPYNE-35S-PmLp8 and pSPYCE-35S-PmWRKY31 vectors were used for BiFC with *Arabidopsis thaliana*. From left to right, the pictures show the yellow fluorescence channel, red fluorescence channel, bright field, and superimposed map. (c) Localization of the PmWRKY31 gene subcellularly. Constructed vector plasmids were transferred into Agrobacterium, and tobacco plants in good growth conditions were selected. A 1-mL syringe without a gun tip used to inject solution into the lower epidermis of tobacco leaves for labeling; plants were incubated in weak light for 2 d after injection. Tobacco leaves were taken, observed and photographed with a confocal laser microscope (Olympus FV1000, excitation light: 480, emitting light: 510). From left to right, the pictures show bright light.

To further confirm the interaction between PmWRKY31 and PmLp8, we performed pull-down experiments (Fig. 2a). We also used BiFC technology to further confirm the protein–protein interactions between PmLp8 and PmWRKY31 of *P. massoniana* (Supplemental Fig. S5). The results showed that PmLp8 interacted with PmWRKY31 in the nuclei of plant cells (Fig. 2b). Subcellular localization of PmWRKY31 also indicated that PmWRKY31 was located in the nucleus (Fig. 2c).

### Analysis of *D. punctatus* behavior on different treatments

**Figure 3:**
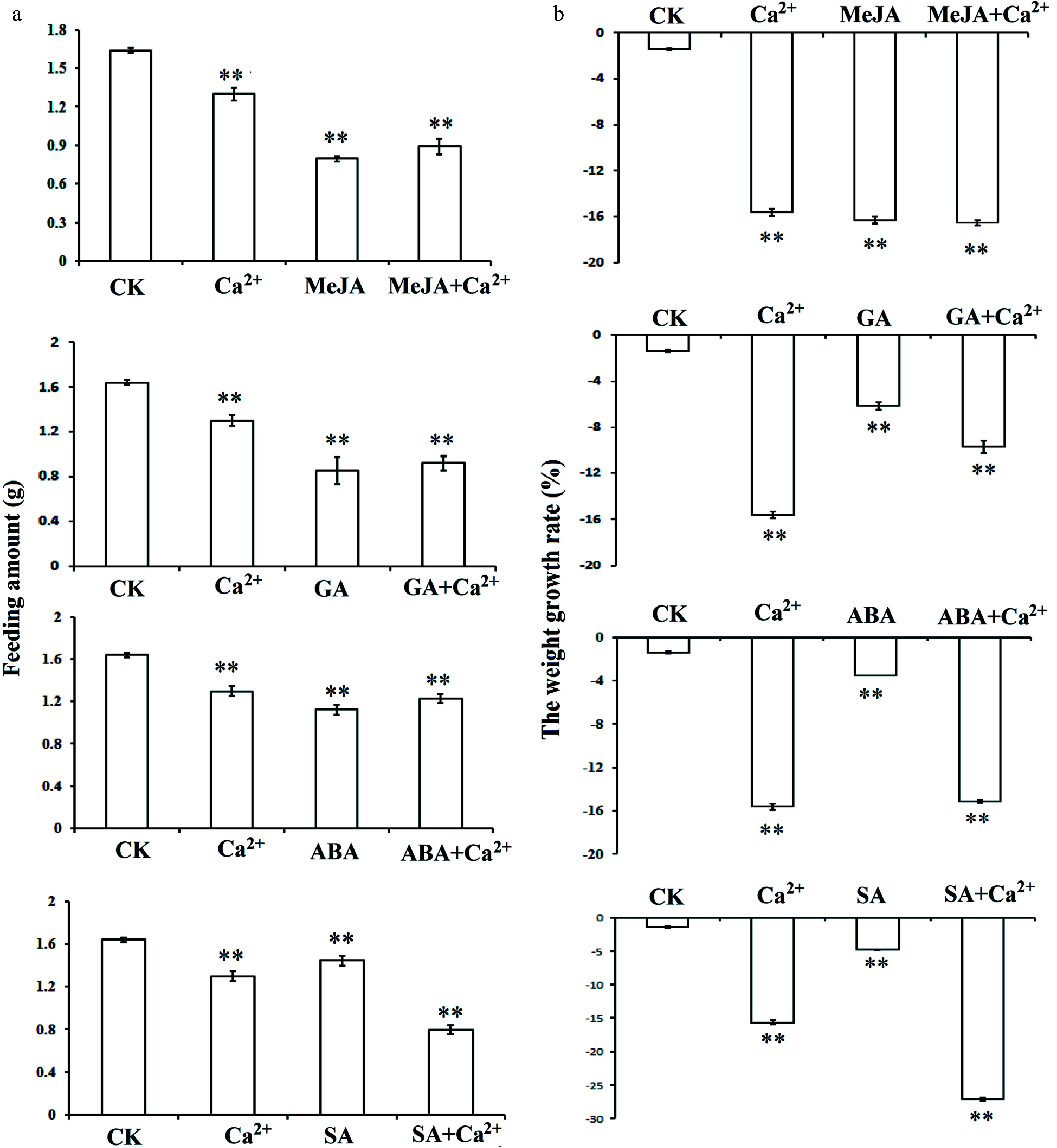
Effects of hormone and Ca^2+^ treatments on *D. punctatus* behavior. The seedlings were treated with different hormones and calcium according to the experimental design. Fresh needles were used to feed the 3rd instar *D. punctatus* with needles sprayed with ddH_2_O water as the control. On the 5th day of feeding, the feeding amount and weight of *D. punctatus* were weighed and calculated according to the corresponding equation. (a) Effects of different treatments on *D. punctatus* feeding; (b) Effects of different treatments on *D. punctatus* weight. Each sample was repeated three times. ** *P* < 0.01, Student's *t*-tests.

The above experiment demonstrated that PmLp8 directly interacted with PmWRKY31. PmLp8 was a kind of calmodulin. We discussed whether hormones and Ca^2+^ might affect the feeding of *D. punctatus*. To this end, we designed different hormone and calcium treatments to verify changes in feeding. The results showed that from the 5th day of treatment, the feeding amount and weight growth rate of *D. punctatus* were significantly decreased when treated with Ca^2+^ alone, each of four hormones alone, and each hormone and Ca^2+^ together (*P* < 0.01). Feeding amounts with, MeJA, GA, and ABA treatments combined with Ca^2+^ were much lower than those with Ca^2+^ treatment alone. Under Ca^2+^ treatment, the feeding amount was slightly higher than that under SA treatment (Fig. 3a). In terms of the weight growth rate, Ca^2+^ treatment alone generated a better effect than MeJA, GA, ABA, or each hormone combined Ca^2+^, but the effect was not as good as that of SA plus Ca^2+^ treatment (Fig. 3b). In general, these four hormones and calcium treatments enhanced the induced resistance of *P. massoniana* to *D. punctatus*.

### Analysis of substances causing antifeeding behavior of *D. punctatus*

**Figure 4:**
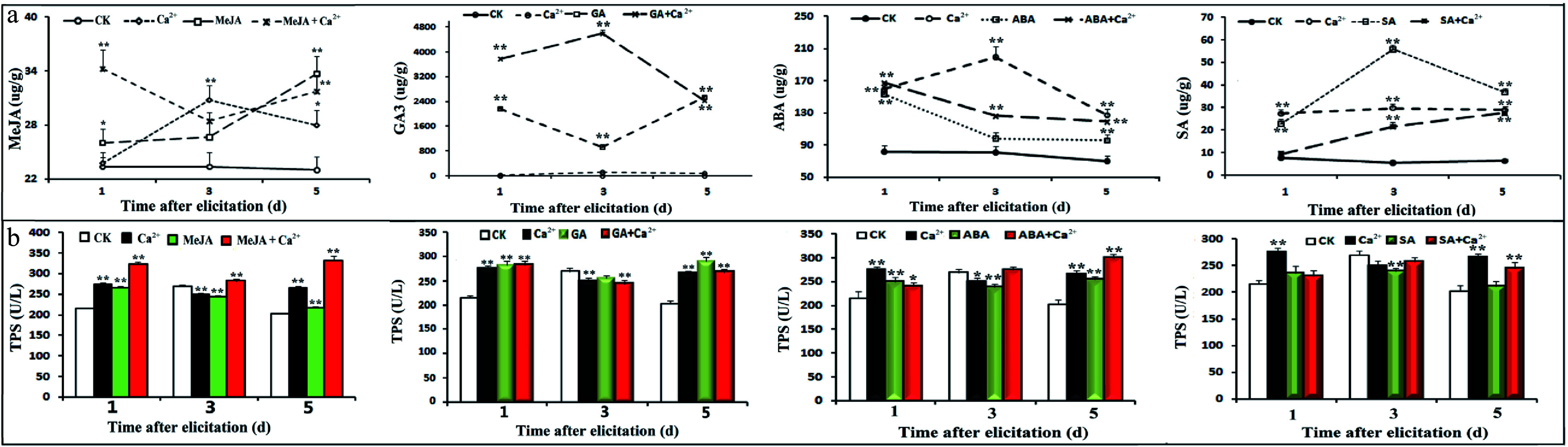
Effects of hormones and Ca^2+^ treatments on hormone and TPS contents. Seedlings were treated with different hormones and calcium according to the experimental design, and samples were collected on days 1, 3 and 5. Four hormones were determined by UPLC-MS-MS. A plant TPS ELISA kit (Shanghai, China) was used to detect of TPSs in different samples according to the manufacturer's instructions. (a) Effects of different treatments on hormones; (b) Effects of different treatments on TPSs.

Based on the above results, we speculated regarding the changes in substances that cause the antifeeding behavior of pine caterpillars. Therefore, several kinds of substances were detected, including hormones, terpenoid synthases, and volatile substances, in needles under different treatments. The results showed that, in general, although the GA content was not significantly increased under Ca^2+^ treatment, the content of related hormones increased to varying degrees (*P* < 0.01) under other treatments. Treatment with hormones and hormones plus Ca^2+^ had the most obvious increasing effect on the corresponding endogenous hormone content (Fig. 4a). Ca^2+^ had a significant increasing effect on the ABA content.

In terms of the TPS content, on the first day of treatment, almost all treatments promoted an increase in the TPS content except for SA and SA plus Ca^2+^. Treatment with MeJA plus Ca^2+^ significantly increased the content of TPS. The effect of SA and SA plus Ca^2+^ treatment on the TPS content was not as good as that of Ca^2+^ treatment alone (Fig. 4b).

**Figure 5:**
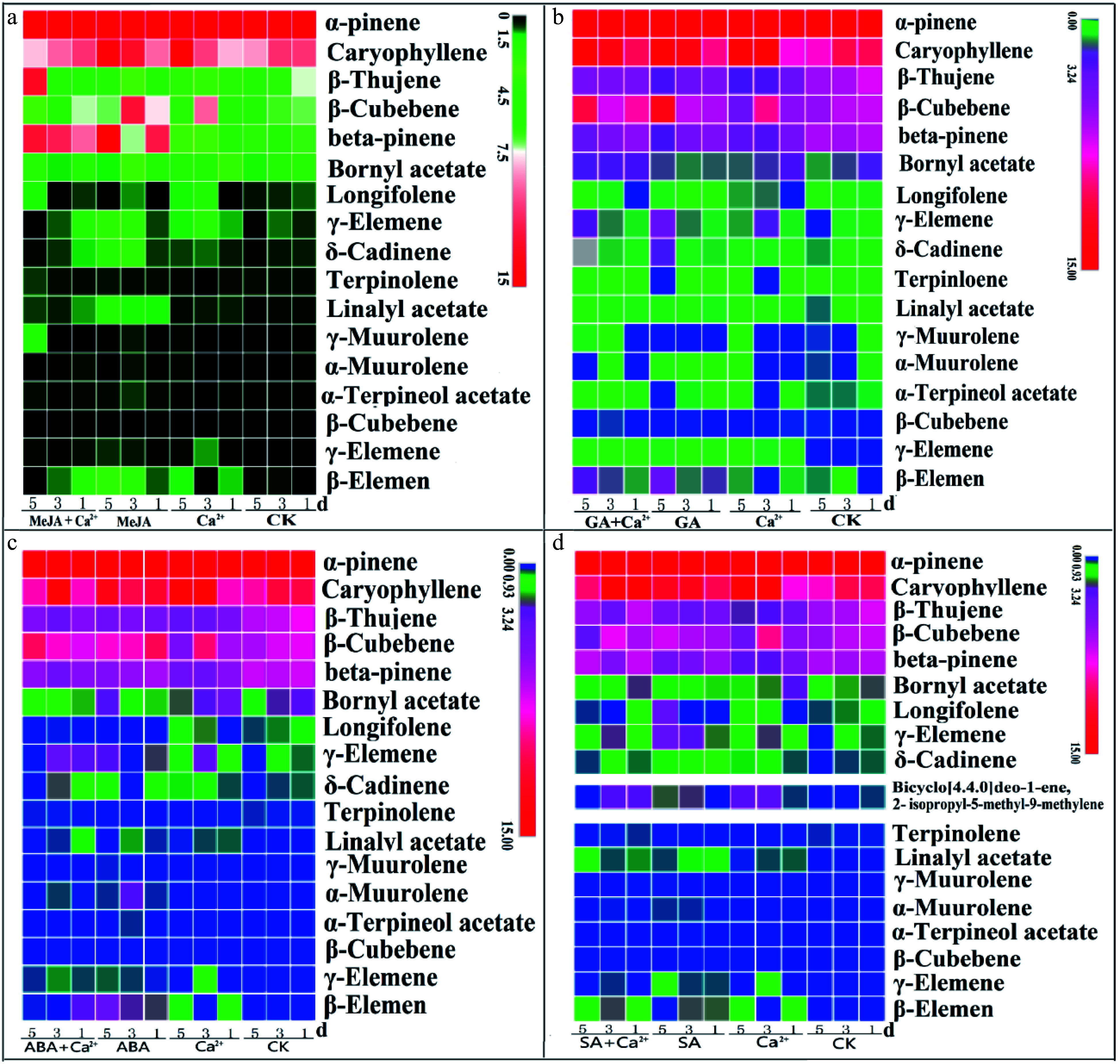
Effects of hormone and Ca^2+^ treatments on volatile substances. Seedlings were treated with different hormones and calcium according to the experimental design. Samples were collected on days 1, 3 and 5. Volatile substances were determined by a SCION SQ and TQ (GC-MS) system. The extract was subjected to GC-MS analysis. (a)−(d) The effects of MeJA, GA, ABA, SA, and Ca^2+^ treatments on volatile substances. Each sample was repeated three times, ** *P* < 0.01, Student's *t*-tests.

We further studied the effects of exogenous hormones and Ca^2+^ treatment on volatile substances. The results showed that the substances affected by the four hormone pathways were basically the same, and a variety of volatile substances were enhanced after treatment. Different treatments could increase the content of volatile substances in needles, especially terpenoids. *β*-Cubebene, caryophyllene, *α*-pinene, *β*-pinene, γ-elemene, and other substances were significantly increased (Fig. 5a−d). The only difference is that exogenous SA plus Ca^2+^ can significantly increase the content of bicyclo[4.4.0]dec-1-ene, 2-isopropyl-5-methyl-9-methylene (Fig. 5d). Above all, the treatments with four kinds of hormones and Ca^2+^ were speculated to increase the content of endogenous hormones, and thus increased terpenoid volatile substances, ultimately enhancing the induced resistance of *P. massoniana* to *D. punctatus*.

### Expression analysis of *PmWRKY31*, *PmLp8* and hormonal signal–related genes after treatment with Ca^2+^ and four kinds of exogenous plant hormones

We evaluated whether *PmWRKY31* can respond to different hormone signaling pathways and is involved in the anti-insect defense of *D. punctatus*. Real-time fluorescence quantitative analysis was performed to test gene expression levels under treatments with the four kinds of hormones and Ca^2+^.

**Figure 6:**
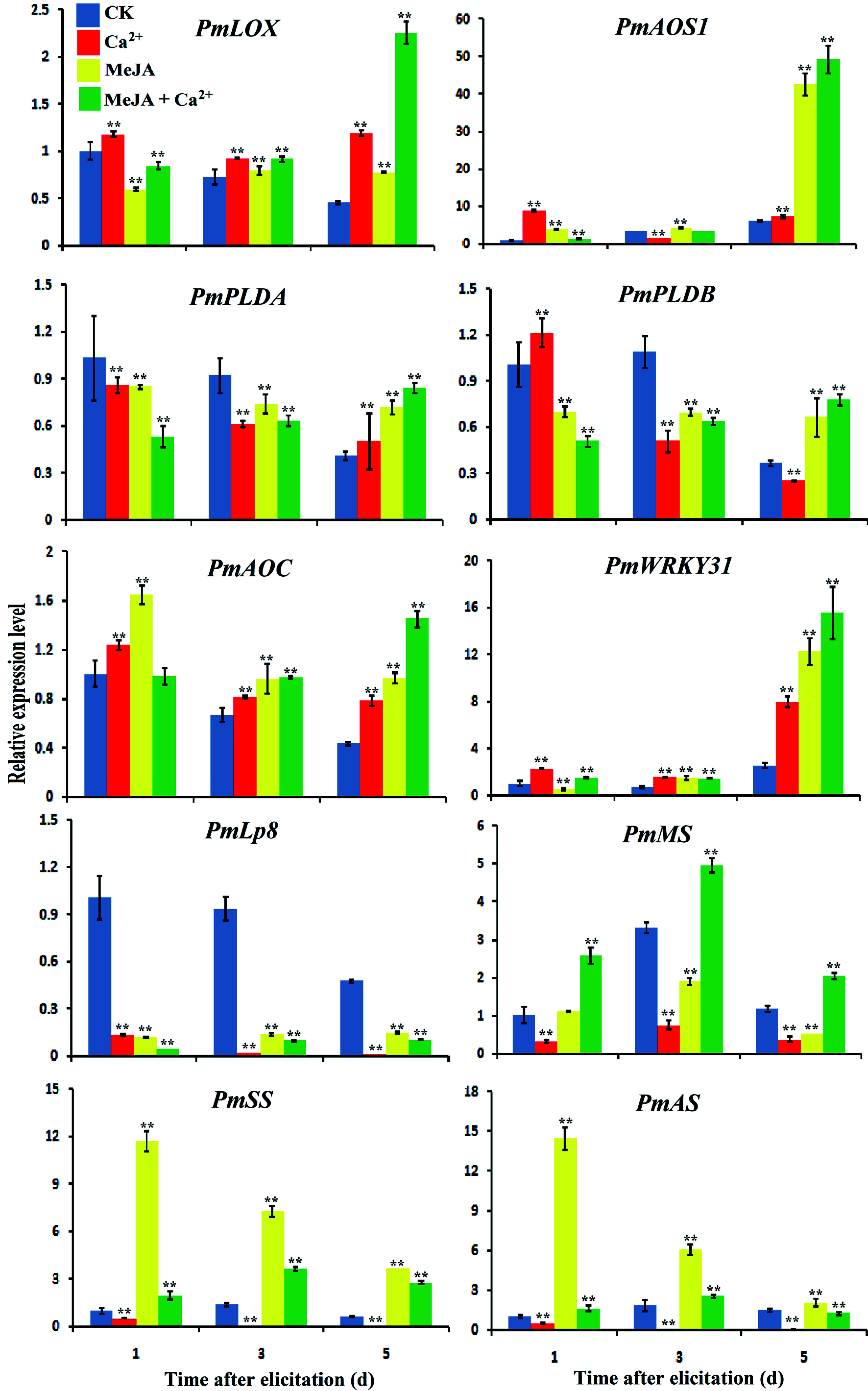
Effects of MeJA and Ca^2+^ treatments on the expression of related genes. Samples were collected on days 1, 3 and 5 of the different treatments according to the experimental design. RNA was extracted using a polyphenol polysaccharide plant RNA extraction kit, cDNA reverse transcription was performed by M-MLV reverse transcriptase, and gene expression analysis was performed using real-time fluorescence quantitative PCR. The gene names were *PmLOX* (Lipoxygenase), *PmAOS1* (Allene oxide synthase), *PmPLDB* (Phospholipase D beta), *PmPLDA* (Phospholipase D alpha), *PmAOC* (Allene oxide cyclase), *PmAS* (Diterpene synthase), *PmSS* (Sesquiterpene synthase), and *PmMS* (Monoterpene synthase). Each sample was repeated three times. ** *P* < 0.01, Student's *t*-tests.

*PmWRKY31*was significantly induced under different treatments, especially on the 5th day of treatment, while *PmLp8* was downregulated under different treatments. Meanwhile, the test results for five genes related to JA synthesis showed that the expression levels of *PmLOX*, *PmAOS1* and *PmAOC* significantly increased at the early stage of treatments, while the expression levels of *PmPLDA* and *PmPLDB* were increased at the later stage of treatments (Fig. 6). Treatment with MeJA and Ca^2+^ promoted the expression of genes involved in the JA pathway. The expression of monoterpene synthesis (MS), sesquiterpene synthase (SS) and diterpene synthase (AS) was not increased by Ca^2+^ treatment alone. The expression of *PmSS* and *PmAS* increased after MeJA treatment. Treatment with MeJA plus GA^2+^ resulted in the most obvious improvement in MS expression (Fig. 6). The Ca^2+^ signaling pathway might be involved in the MeJA metabolic pathway through positive regulation of *PmWRRKY31* to improve resistance to *D. punctatus*.

GA improved the expression of *PmLp8*, *PmGGPS*, and *PmKS* after 5 days of treatment. Treatment with GA and Ca^2+^ downregulated the expression of *PmKAO*, *PmGPS*, *PmKO*, and *PmGID1*. *PmWRKY31* expression was highest under Ca^2+^ treatment (Supplemental Fig. S6). GA treatments promoted the expression of three terpenoid synthase genes. *PmWRKY31* might be involved in responding to GA signaling and may be positively regulated in this process.

Treatment with of Ca^2+^ and ABA plus Ca^2+^ improved *PmWRKY31*, expression but downregulated *PmICS* expression in the ABA synthesis pathway, the terpene synthase gene and *PmLp8*. ABA increased the expression of only *PmWRKY31* and *PmLp8* (Supplemental Fig. S7a). The results under SA treatment were similar to those under ABA treatment (Supplemental Fig. S7b). Overall, *PmWRKY31* might respond to hormone and Ca^2+^ signals to improve the induced resistance of *P. massoniana* to *D. punctatus*.

### Verification of the function of *PmWRKY31*

**Figure 7:**
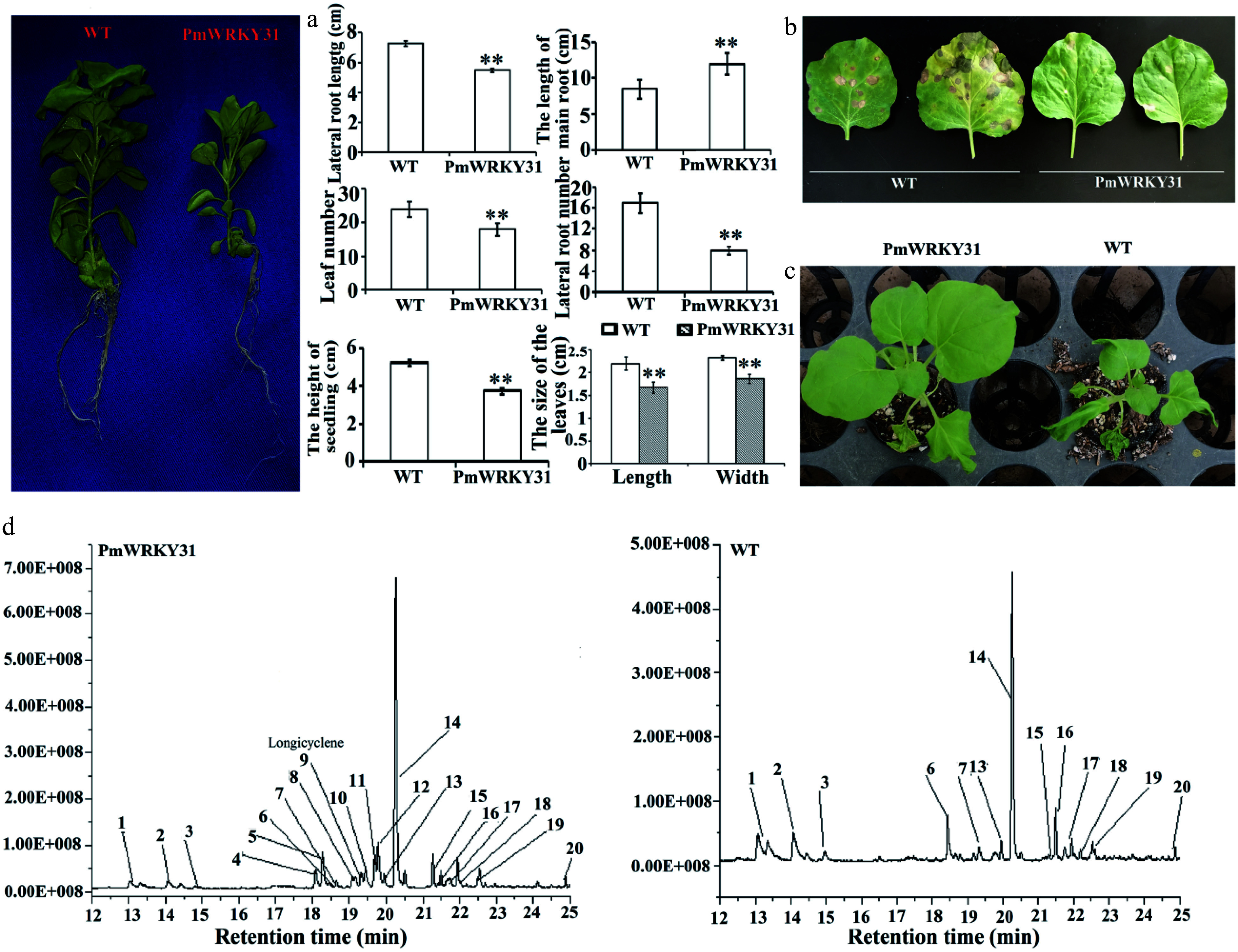
Function verification of *PmWRKY31*. (a) Effects of a transgene on tobacco plants. (b) Effects of a transgene on disease resistance. (c) Effects of a transgene on drought tolerance. (d) Effects of a transgene on volatiles. Each sample was repeated three times. ** *P* < 0.01, Student's *t*-tests.

To further study the function of the *PmWRKY31* gene, we conducted overexpression experiments. The root length of *PmWRKY31* overexpressing plants was significantly greater than that of the control, and the height, number of lateral roots, lateral root length, leaf number, and leaf size were significantly lower than those of the control (Fig. 7a). Although the biomass of *PmWRKY31* transformation plants was smaller than that of the wild-type plants, their resistance to tobacco anthracnose (Fig. 7b), and drought (Fig. 7c) was significantly stronger than that of the wild-type plants. Similarly, the resistance of *PmWRKY31* transformation plants against *Helicoverpa assulta* Guenee was also significantly improved. We tried to feed the tobacco leaves of *PmWRKY31* transformation plants and wild tobacco plants to *D. punctatus*, but the insect accepted neither of them, possibly due to its feeding characteristics. In summary, the above experiments further confirm that the *PmWRKY31* gene plays a key role in insect resistance as well as resistance to diseases and abiotic stresses.

Further analysis of the volatile substances showed that in *PmWRKY31* transformation plants, the concentrations of more than 10 volatile substances, especially 2,6-diisocyanato-1-methyl-benzene (4), tolylene-2,4-diisocyanate (5), 2,4-diaminotoluene (8), longicyclene (9), 6-Aminoindolone (10), 5-methyl-2-benzimidazolone (11), N-methyl-1,3-benzoxazole-2-amine (12) and longifolene changed (Fig. 7d; Supplemental Table S2). These results indicated changes in terpene concentrations, which might suggest that *PmWRKY31* regulates the expression of downstream genes, thereby increasing terpenoid concentrations to improve insect resistance.

## DISCUSSION

### *PmWRKY31* is a key regulator of insect feeding–induced defense responses

After sequencing, we obtained three WRKY genes that were associated with the insect resistance from *P. massoniana*. All of them contained the specific and conserved WRKY domains that specifically bind to W-boxes^[36,37]^. Clustering analysis can classify WRKY genes according to the characteristics of their zinc finger domains^[38]^.

Signaling molecules such as MeJA, SA, GA, and ABA play important roles in plant defense against insects^[3,7,9,10]^. We examined the feeding characteristics of pine caterpillars and tested hormones, gene expression, and terpenoids under MeJA, SA, GA, and ABA to confirm whether *PmWRKY31* regulated these signaling pathways. The results of these all indicated that *PmWRKY31* plays important roles in the insect resistance of *P. massoniana*. First, high *PmWRKY31* expression was induced by treatments of MeJA, GA, ABA, SA and Ca^2+^. Second, *PmWRKY31* changed the concentrations of volatile substances in tobacco and improved their insect resistance, disease resistance, and drought resistance. Third, *PmWRKY31* interacted with the Ca^2+^ signaling protein *PmLP8* and changed concentrations of several kinds of hormones and TPSs.

### Possible mechanism of the interaction between *PmWRKYY31* and Ca^2+^ signaling

Ca^2+^ signaling is involved in plant defense against herbivorous insects, and insect feeding can activate Ca^2+^ signaling^[3,39−41]^. However, in the interaction between *D. punctatus* and *P. massoniana*, the relationship between Ca^2+^ signaling and the defense response of *P. massoniana* induced by *D. punctatus* is still unknown. We found that treatment of exogenous Ca^2+^ increased the concentrations of TPSs, hormones, and volatile substances in *P. massoniana* and significantly upregulated *PmWRKY31*.

Treatment with MeJA plus Ca^2+^ increased *PmLp8* expression, while treatments with other hormones plus Ca^2+^ decreased *PmLp8* expression. Therefore, we speculated that *PmWRKY31* negatively regulated PmLp8 to improve resistance to *D. punctatus*. In this process, MeJA and the other three hormones have different regulatory mechanisms. Downregulation of *Lp8* genes was found in a study on the defense of *P. massoniana* against *Bursaphelenchus xylophilus*^[42]^. Ca^2+^ phosphorylation regulates the MeJAZ8–WRKY51 network to improve the insect resistance of plants^[41]^. Ca^2+^ in chloroplasts can positively regulate activity of *MPK3/MPK6*^[43]^. Calcium-dependent protein kinases and mitogen-activated protein kinases (MAPKs) can positively regulate the pathogen defense of *Arabidopsis*^[44]^. WRKY genes regulate MAPK genes to improve the insect resistance of rice^[7,29,45,46]^. In this study, we demonstrated that PmWRKY31 and PmLp8 can interact both in vivo and in vitro through assays of yeast two-hybrid, BiFC, and pull-down.

### *PmWRKY31* plays an important regulatory role in the insect resistance mechanism of *P. massoniana* against *D. punctatus*

WRKY transcription factors play key roles in hormone signaling pathways and in the regulation of plant resistance genes^[26,37,47−49]^. We found that the application of exogenous MeJA, SA, ABA, or GA increased the MeJA, SA, ABA, or GA concentrations in needles of *P. massoniana* and significantly upregulated the *PmWRKY31* gene, indicating that the *PmWRKY31* gene improved the insect resistance of *P. massoniana* through participating in the signaling pathways of MeJA, SA, ABA, and GA. Transformation of *OsWRKY13* and *OsWRKY30* in rice enhances the resistance of rice plants to leaf blight and rice blast^[50,51]^. *OsWRKY53* can positively regulate SA biosynthesis^[29]^. *OsWRKY13* can activate ICS1, a key enzyme in SA biosynthesis, and transgenic seedlings of *OsWRKY13* had high SA accumulation^[50]^. *ThWRKY4* can increase the tolerance of ABA-treated *Tamarix hispida*^[52]^. Based on the above evidence and our experimental results, we can conclude that the targets of *PmWRKY31* may be MeJA, GA, ABA, and SA biosynthesis–related genes, including LOX/AOS1/AOC^[1,12]^, GGPS/KS, and ICS^[53,54]^. However, it remains unclear whether *PmWRKY31* directly or indirectly binds to the W-box of these gene promoters, further study is needed to clarify.

### *PmWRKY31* plays an important regulatory role in the biosynthesis of terpenoids for the insect resistance of *P. massoniana*

TPSs play special roles in insect resistance^[33,34]^. They are also involved in the biosynthesis of phytoalexin and regulation of some hormonal substances in plant defense responses^[20]^. TPSs genes have been cloned from more than 40 species of plants^[20,21]^.

In constitutive and induced plant defenses against herbivorous insects, biosynthesis of terpenes are regulated by a variety of hormones (including endogenous and exogenous hormones). The defensive metabolites of GA and diterpene in rice plants have been studied in detail, and the results indicate that *OsCPS1* downstream of the GA pathway was involved in the biosynthesis of terpenoids^[34]^. Higher concentration of SA significantly improve the insect resistance of plants^[23,24]^. WRKY1 band to the W-box of the CAD1-A promoter, activated the expression of *CAD1-A*, and regulated secondary metabolism. This gene can also interact with MeJA and GA signaling molecules to coordinate the biosynthesis and volatilization of terpenes^[19]^. Our study showed that treatments with MeJA, GA, SA, ABA, and Ca^2+^ increased the concentrations of TPSs, volatile terpenes and the expression of the MS, SS, and AS genes. Transgenic tobacco of *PmWRKY31* showed that the concentration of sesquiterpenes was significantly increased. Therefore, *PmWRKY31* might indirectly affect the concentration of terpenoids in plants, thereby improving the insect resistance of plants.

## CONCLUSIONS

**Figure 8:**
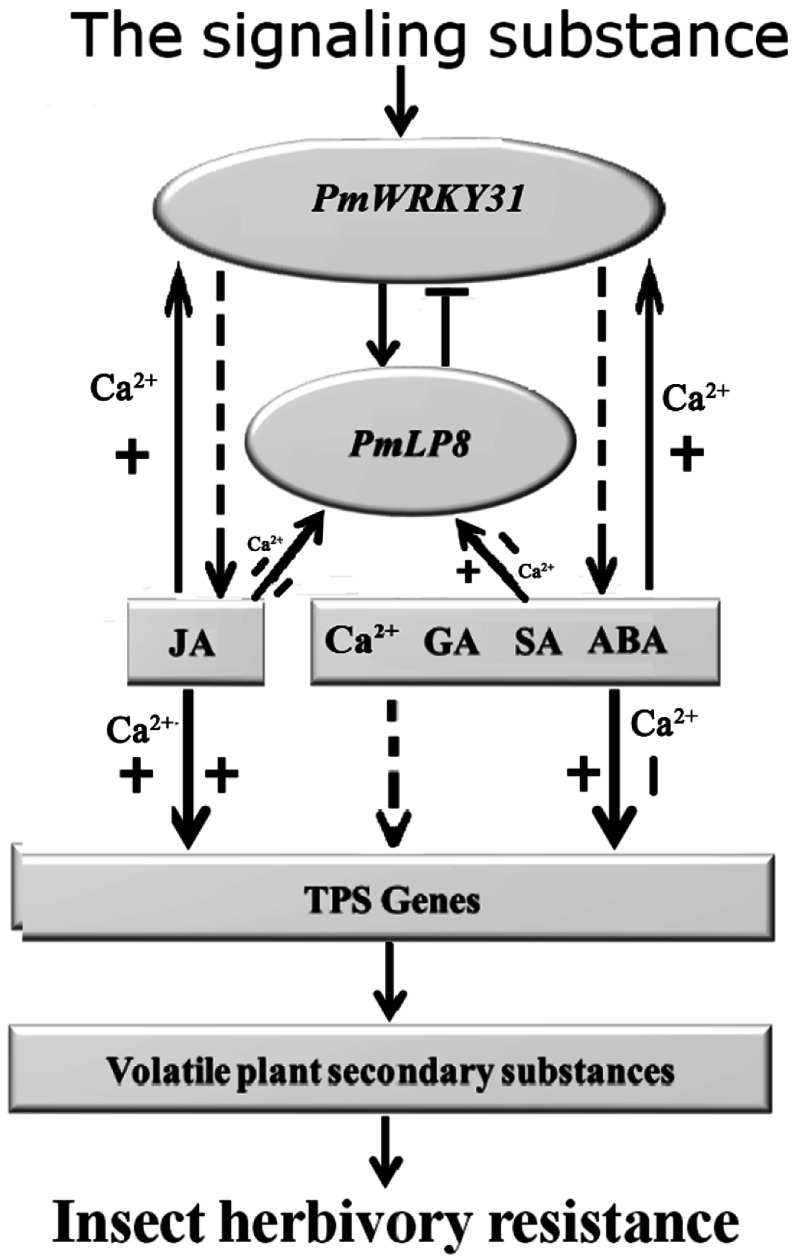
Preliminary model of improving the *PmWRKY31* gene and *PmLp8* gene interaction to regulate hormone and calcium signaling pathways to enhance resistance to *D. punctatus*. The application of exogenous signaling substances was able to rapidly initiate the expression of *PmWRKY31*, and increase the downstream hormone signals and key gene responses of the terpene synthesis pathway by regulating the *PmLp8* gene, thereby increasing the content of endogenous MeJA, GA, SA, ABA and terpene synthase and volatiles in *P. massoniana* to promote the ability to resist *D. punctatus*. All treatments strongly induced *PmWRKY31* expression. MeJA, MeJA+Ca^2+^, GA, ABA, and SA positively regulated *PmLp8* and terpene synthase genes, while treatment with GA, ABA, and SA with Ca^2+^, and Ca^2+^ negatively regulated these genes. Arrows represent negative or regulation; barred lines represent negative regulation; arrows with "+" represent positive regulation, and arrows with "−" represent negative regulation.

In summary, treatments with exogenous hormones and Ca^2+^ increase the concentrations of endogenous hormones and TPSs of *P. massoniana*. PmWRYK31 could interact with PmLP8. All treatments in this study strongly induced the expression of *PmWRKY31*. Most treatments inhibited *PmLP8* expression except for GA, ABA, and SA treatments. Treatment with MeJA, MeJA plus Ca^2+^, GA, ABA, and SA upregulated the expression of terpenoid synthase-related genes, while other treatments downregulated their expression. *PmWRYK31* may be involved in the regulation of gene expression in several hormone signaling pathways, the expression of terpenoid synthase genes. As a result, the concentration of terpenoid substances were correspondingly improved to enhance the resistance of *P. massoniana* to *D. punctatus* (Fig. 8).

## MATERIALS AND METHODS

### Plant materials

The seeds of *P. massoniana* were F1 hybrids (No. 17-243) with 229D as the female parent and 228D as the male parent. This experiment was performed in full accordance with the 'Seed zones of Chinese forest tree-Seed zones of *P. massoniana'* (GB 8822.6-1988) of the National Forestry and Grassland Administration. Tobacco plants were grown from *Nicotiana benthamiana* seeds. The seeds were stored at 4 °C until further use.

### Plant cultivation

The seeds were sown in yellow soil for germination. When the seedlings grew to 5 cm tall, they were transplanted into nonwoven bags with a diameter of 12−15 cm. The light medium was formulated with 45%−60% peat or coconut chaff, 20%−30% carbonized rice chaff, 8%−9.5% perlite, 1% calcium superphosphate, and 10%−15% peat soil. The buds were planted in the breeding nursery for pine seedlings of the Guangxi Academy of Sciences (Nanning, Guangxi, China). Healthy 1-year-old seedlings of *P. massoniana* with good growth, the same height, no insect damage, and no mechanical damage were selected for as experiments.

Tobacco was cultivated in trays. After the seeds were scattered on top of the soil, the seeds were covered with a light substrate, watered thoroughly and placed in a light incubator under an 8/12 h light/dark cycle (1,200 lx light intensity, 75% air humidity). When seedling height of the tobacco was approximately 5 cm, the plants were transferred to pots and cultivated in the greenhouse. One plant was planted in one pot with normal management.

### Experimental *D. punctatus*

*D. punctatus* (fifth-instar larvae) was collected from a *P. massoniana* seed orchard (Ningming and Nanning, Guangxi, China). *D. punctatus* was collected in a transparent nylon net cage (25 cm × 30 cm) and cultured in a constant temperature and light incubator (temperature: 28 ± 0.5 °C, relative humidity: 75 ± 5%, light : dark = 16:8). Fresh *P. massoniana* needles were used as fodder. The fresh fodder was changed every two days and the dung in the cage was cleaned until the larvae cocooned and pupated. On day 16 of cocooning, the cocoons were cut open with surgical scissors, the pupae were removed, and the pupae were raised under the same temperature, humidity, and light conditions. Fresh needles were also used as fodder during adult eclosion. The pupae were first used in experiments at the age of 3 d.

### Plant treatments

The following treatment solutions were prepared: 75 mg/L ABA, 75 mg/L ABA + 100 mg/L CaCl_2_, 50 mg/L SA, 50 mg/L SA + 100 mg/L CaCl_2_, 100 mg/L MeJA, 100 mg/L MeJA +100 mg/L CaCl_2_, 150 mg/L GA, 150 mg/L GA + 100 mg/L CaCl_2_, 100 mg/L CaCl_2_ (dissolved in 50 mM phosphate buffer, pH 8.0), and ddH_2_O water treatment as the control (CK). Distilled water was used as a control. Before use, 0.01 (v/v) Tween-20 was added to the solutions, and the treatment solutions were evenly sprayed on the *P. massoniana* seedlings once every 1 d for 5 d at 200 mL/treatment. Ten *P. massoniana* seedlings with uniform growth were used for each treatment. Mature needles were collected 1 d, 3 d, and 5 d after the treatment ended. The collected needles were divided into two groups. One group was immediately tested to measure volatile substances, and the other group was immediately stored in liquid nitrogen, transferred to the laboratory, and stored at −80 °C for future use. ABA, SA, MeJA, and GA (purity > 95%) were purchased from Sigma-Aldrich.

### *D. punctatus* feeding treatment

An insect incubator was used for each *D. punctatus* feeding treatment, and 15 *D. punctatus* of the same size were selected for each treatment. They were placed in plastic boxes (length × width × height = 20 cm × 15 cm × 7 cm). One box was used for each treatment, and each treatment was repeated three times. On day 5, the *D. punctatus* samples were observed. The effects of different treatments on *D. punctatus* were investigated based on their food intake and growth conditions. The food intake calculation formula for larvae was as follows:

Feeding amount = (amount of feed input − amount of residual feed) × (1 − water loss rate),

water loss rate = (weight of needle leaves in second day/weight of fresh needle leaves in first day) × 100%;

Weight growth rate = [(insect weight on day 5 − insect weight on day 1)/insect weight on day 1] × 100%.

The treatments were carried out in net houses.

### RNA extraction and reverse transcription

RNA was extracted according to the instructions of the RNA Isolation Kit for polyphenol- and polysaccharide-rich plants (Tiangen Biotech, Beijing, China). The reverse transcription primer was Oligo(dT)_18_: 5'-GGCCACGCGTCGACTAGTAC(T)18-3'. Specific cDNA was synthesized according to the instructions of M-MLV reverse transcriptase. After completion, 4 μL of the polymerase chain reaction (PCR) product of each treatment was used for agarose gel electrophoresis, and the cDNA concentration of each treatment was measured using a UV spectrophotometer and then diluted to the same concentration.

### Isolation and characterization of gene expression from cDNA

Based on transcriptome data (transcriptome data uploaded to NCBI GEO, accession number GSE72294.) and protein–protein interactions, the full lengths of *PmWRKY31 *and *PmLp8* were obtained. Primer 5 software was used to design full-length primers to amplify these genes (Supplemental Table S2). The PCR products were cloned into the pMD19-T vector (TaKaRa) and sent to Sangon Biotech (Shanghai, China) for sequencing.

### Bioinformatics analysis

WoLFPSORT software was used to predict the subcellular localization of proteins. The amino acid sequences of the proteins were constructed with ClustalX^[55]^ and MEGA4.1 software^[56]^. The online software NCBI, SMART^[57]^, and Motif Scan^[58]^ were used to analyze the functional domains of genes. Protein–protein interactions were predicted using the STRING database (https://string-db.org/cgi)^[59]^. Transcriptome data and QuickGO (www.ebi.ac.uk/QuickGO)^[60]^ were used to predict gene function, and the Kyoto Encyclopedia of Genes and Genomes (KEGG) data were used for metabolic pathway analysis^[61]^.

### Subcellular localization

The constructed pBWA(V)HS-PmWRKY31-GLosgfp vector plasmid was transferred into Agrobacterium. After Agrobacterium-coated plates were incubated at 30 °C for 2 d, Agrobacterium was inoculated into 10 mL of YEB liquid medium and resuspended in 10 mM MgCl_2_ suspension (containing 120 µM AS), and the optical density measured at a wavelength of 600 nm (OD_600_) was adjusted to approximately 0.6. The suspension was injected into the epidermis of a tobacco leaf with a 1-mL syringe (needle removed). After injection, the tobacco plants were cultured under low light intensity for 2 d. Next, the tobacco leaves were collected and imaged directly under a laser confocal microscope (FV10-ASW, OLYMPUS, Shenzhen, China). In the subcellular colocalization experiment, although the nuclear marker and the plasmid vector were simultaneously transferred to Agrobacterium before plating and incubation, all other steps were the same.

### Transgene expression

Sterile tobacco seedlings were induced using mature tobacco embryos. The plasmid pBI121-PmWRKY31 was constructed and transferred to Agrobacterium EHA105 and stored in a −80 °C freezer. The Agrobacterium-mediated transformation of tobacco seedlings followed the steps described by Yu et al.^[62]^. The cetyltrimethylammonium bromide (CTAB)-based method was used to extract DNA from tobacco seedlings, and primers specific for resistance genes (Supplemental Table S1) were used to amplify and detect the presence of PmWRKY31 in tobacco seedlings using the conventional PCR method. Transgenic lines were screened from the F3 generation of tobacco plants transduced with PmWRKY31, morphological indicators were observed, and hormones, volatile substances, and resistance were determined.

### Yeast two-hybrid assay

#### Construction of the cDNA library

After RNA extraction, cDNA was synthesized and purified. The purified cDNA was homogenized and further purified. cDNA was digested using the restriction endonuclease SfiI. After the digested cDNA was passed through CHROMA SPIN-1000-TE columns, an appropriate amount of cDNA was ligated into the pGADT7-SfiI vector (TaKaRa, China) at 12 °C using the DNA ligation kit (O/N linked) and purified to obtain the primary cDNA library, which was electroporated into HST08 competent cells. Ten large Luria-Bertani (LB) agar plates (24.5 cm × 24.5 cm) were coated with these cells and cultured overnight at 37 °C, and the number of clones obtained after the transformation was monitored.

#### Yeast two- and four-hybrid assays

Five micrograms of the bait plasmid was transformed into Y187 yeast, and 100 SD/Leu plates were coated with yeast and cultured at 30 °C for 3 d. The pGBKT7-PmWRKY31 plasmid vector was constructed, and the bait plasmid was transformed into the Y2HGold strain to obtain the bait strain. The expression of the exogenous proteins in the bait strain was detected by western blotting.

Two-hybrid screening: Bait-Y2HGold strains were cultured using the streak plate method for 3 d. Colonies were selected and cultured in SD/-Trp broth and mated to the Y187 yeast library. A small amount of suspension was diluted to 1/10, 1/100, 1/1,000, and 1/10,000, and 100 μL of the diluted suspension was used to coat 100-mm monitoring plates. The suspension was coated onto 50 to 55 SD/-Trp/-Leu/X-a-Gal/Aba plates for two-hybrid screening.

Four-hybrid screening: Blue colonies were counted and inoculated onto SD/-Ade/-His/-Leu/-Trp/X/A plates with a pipette tip and cultured at 30 °C for 5 d. The positive bacterial strain was used as a template for PCR amplification. The AD plasmids in the positive clones were detected, and the amplified products were detected by electrophoresis and sequenced.

### Bimolecular fluorescence complementation (BiFC)

*PmLp8* and *PmWRKY31* were separately cloned into pSPYNE-35. Proteases were isolated from 3-to-4-week-old Arabidopsis plants with robust growth, transfected by the polyethylene glycol (PEG) method, and observed under a confocal microscope (FV10-ASW, OLYMPUS).

### Pull-down assay

Primers for *PmLp8* and *PmWRKY31* were designed in CmSuite8 software (Supplemental Table S2). The target gene fragment was amplified by high-fidelity PrimeSTAR DNA Polymerase with the WRKY plasmid as the template. Five micrograms of the pGEX-4T-1 vector was digested with *XhoI* and *BamHI* to recover the target fragment. Ligation was performed according to the manufacturer's instructions for the ClonExpress II One Step Cloning Kit (Vazyme Biotech). The ligation products were transformed into Stbl3 competent cells and screened on LB plates containing kanamycin and ampicillin antibiotics (100 μg/mL). Positive clones were confirmed by sequencing.

The fusion protein was subjected to prokaryotic expression, pull-down, western blot, and electrophoresis experiments, transfererd onto membranes, incubated with antibodies, and exposed according to the manufacturers' instructions. GST antibody and HIS antibody were purchased from TRANS (Shenzhen, China), and HRP-labeled goat anti-mouse IgG was purchased from CWBiotech (Beijing, China).

### Real-time fluorescence-based quantitative PCR

Key genes involved in metabolic pathways, such as ABA, GA, MeJA, SA, and terpene biosynthetic pathways, which might interact with WRKY genes, were selected. Primer 5 software was used to design primers for fluorescence-based quantitative PCR, and *PmCYP* (CYP: cyclophilin) was used as a reference gene (Supplemental Table S3)^[63]^. The LightCycler 480II PCR system was programmed according to the instructions of the SYBR Premix Ex Taq II (Perfect Real Time) kit (TaKaRa, China) to conduct fluorescence-based quantitative PCR. All experiments were repeated three times. The relative expression level was calculated according to the $2^{-\Delta\Delta} $^Cᴛ^ method^[64]^, and Microsoft Excel was used for plotting.

### Determination of volatile substances

Volatile substances were determined by a SCION single-quadrupole (SQ) and triple-quadrupole (TQ) gas chromatography mass spectrometry (GC-MS) system. After collecting the needles from the plant, they were placed into fresh bags and stored in the refrigerator at 4 °C. Each treatment (0.5 g) was placed in a 10-mL-headspace bottle, and an appropriate amount of anhydrous sodium sulfate was added. After an aged solid-phase microextraction fiber was inserted into the bottle, the bottle was sealed and placed into a 75 °C thermostat bath for 15 min. The extract was subjected to GC-MS analysis. Each experiment was run three times (technique duplicates). All the needles were measured in one day.

### Determination of hormones

First, 0.1 g of sample was ground in liquid nitrogen, and added to 1 mL methanol solution (methanol : water : formic acid = 75:20:5). After 16 h of extraction in darkness, the supernatant was collected by centrifugation. The above steps were repeated once, and the supernatant was collected and combined with the previously obtained supernatant. The combined supernatant was concentrated and evaporated at 35 °C until no residual methanol remained (changed color). Then, 500 µL of ethyl acetate was added to the remaining aqueous phase for extraction, and the upper, ester phase was collected. This step was repeated three times, and the obtained ester phases were combined. The combined ester phase was concentrated and evaporated at 35 °C until dry. The precipitate was dissolved in 200 µL of methanol, filtered through a 0.22-nm organic membrane, and tested by a liquid chromatography (LC)–MS system (6460 Triple Quad LC/MS, Agilent, USA) with a C18 column (2.1 mm × 100 mm, 1.9 μm). According to the plotted standard curve and the peak area of the substance in the sample tested, the concentration of the substance in the sample was calculated.

### Detection of terpene synthases (TPSs)

A total of 0.1 g of the mixed sample was added to 900 μL of phosphate-buffered saline (1 : 9 weight : volume ratio) and fully ground to homogenate on ice. After the homogenate was centrifuged at 5000 × g for 5−10 min, the supernatant was collected for detection. A plant TPS ELISA kit (Shanghai, China) was used to detect TPSs in different samples according to the manufacturer's instructions. The microplate reader was purchased from Epoch (BioTek, USA).

## SUPPLEMENTARY DATA

Supplementary data to this article can be found online.
