# Supplementary material for: Roles of hormones, calcium and PmWRKY31 in the defense of Pinus massoniana Lamb. against Dendrolimus punctatus Walker
Source: For Res (Fayettev). 2021 Dec 3;1:21. doi: 10.48130/FR-2021-0021 (PMC11524255; doi:10.48130/FR-2021-0021)
Supplement: Supplementary file 1 — Supplementary data to this article can be found online. [file FR-2021-0021-S1.zip › 10.48130_FR-2021-0021-Suppl-FigureS4.pdf]

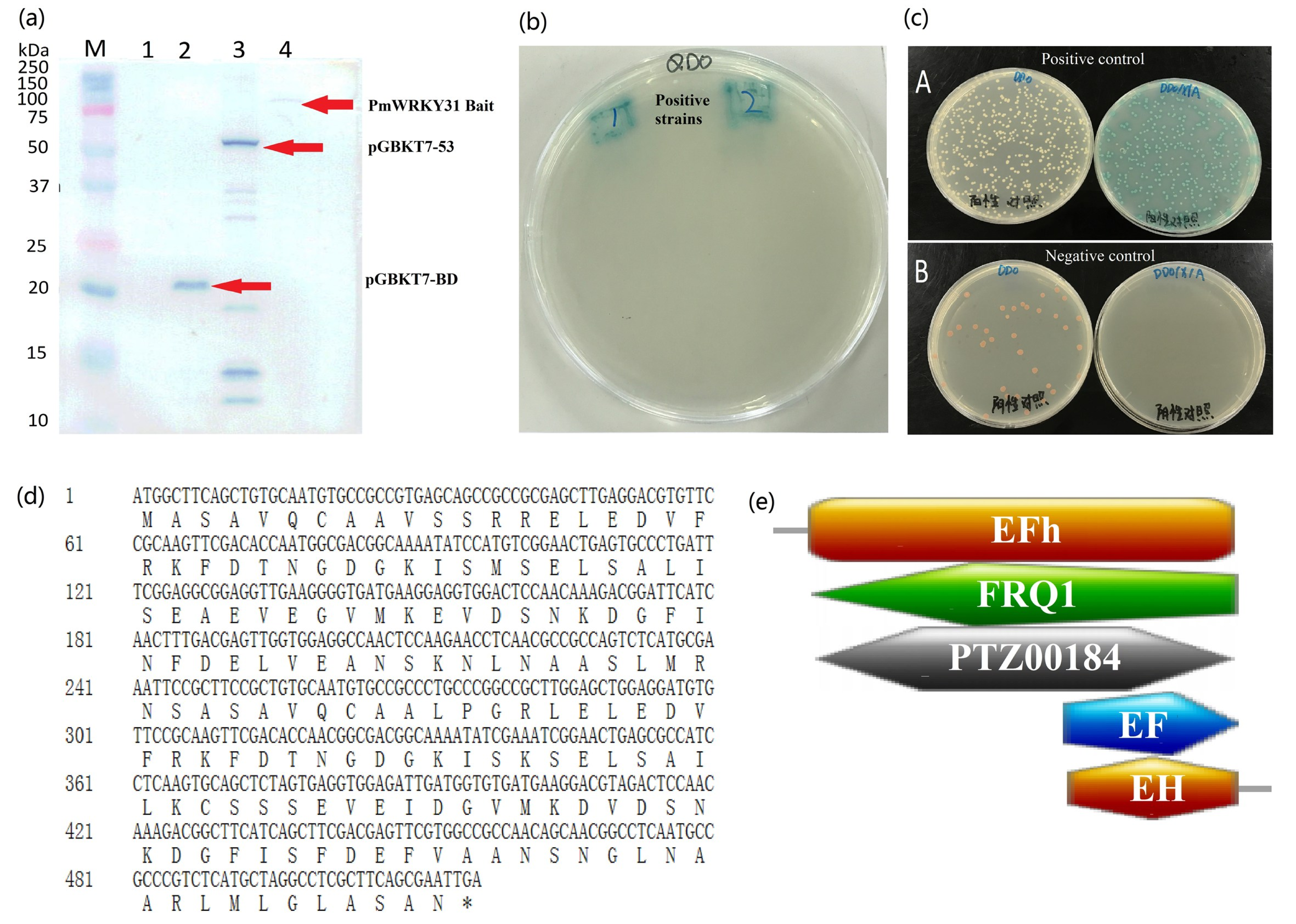
(a)
kDa
M
1
2
3
4
250
150
100
75
50
37
25
20
15
10
PmWRKY31 Bait
pGBKT7-53
pGBKT7-BD
(b)
QDO
1
2
Positive strains
(c)
Positive control
A
DDO
DDO/X/A
Negative control
B
DDO
DDO/X/A
(d)
1 ATGGCTTCAGCTGTGCAATGTGCCGCCGTGAGCAGCCGCCGCGAGCTTGAGGACGTGTTC
M A S A V Q C A A V S S R R E L E D V F
61 CGCAAGTTCGACACCAATGGCGACGGCAAAATATCCATGTCGGAACTGAGTGCCCTGATT
R K F D T N G D G K I S M S E L S A L I
121 TCGGAGGCGGAGGTTGAAGGGGTGATGAAGGAGGTGGACTCCAACAAAGACGGATTCATC
S E A E V E G V M K E V D S N K D G F I
181 AACTTTGACGAGTTGGTGGAGGCCAACTCCAAGAACCTCAACGCCGCCAGTCTCATGCGA
N F D E L V E A N S K N L N A A S L M R
241 AATTCCGCTTCCGCTGTGCAATGTGCCGCCCTGCCCGGCCGCTTGGAGCTGGAGGATGTG
N S A S A V Q C A A L P G R L E L E D V
301 TTCCGCAAGTTCGACACCAACGGCGACGGCAAAATATCGAAATCGGAACTGAGCGCCATC
F R K F D T N G D G K I S K S E L S A I
361 CTCAAGTGCAGCTCTAGTGAGGTGGAGATTGATGGTGTGATGAAGGACGTAGACTCCAAC
L K C S S S E V E I D G V M K D V D S N
421 AAAGACGGCTTCATCAGCTTCGACGAGTTCGTGGCCGCCAACAGCAACGGCCTCAATGCC
K D G F I S F D E F V A A N S N G L N A
481 GCCCGTCTCATGCTAGGCCTCGCTTCAGCGAATTGA
A R L M L G L A S A N *
(e)
EFh
FRQ1
PTZ00184
EF
EH
