# Supplementary figures and images for: Roles of hormones, calcium and PmWRKY31 in the defense of Pinus massoniana Lamb. against Dendrolimus punctatus Walker
Source: For Res (Fayettev). 2021 Dec 3;1:21. doi: 10.48130/FR-2021-0021 (PMC11524255; doi:10.48130/FR-2021-0021)

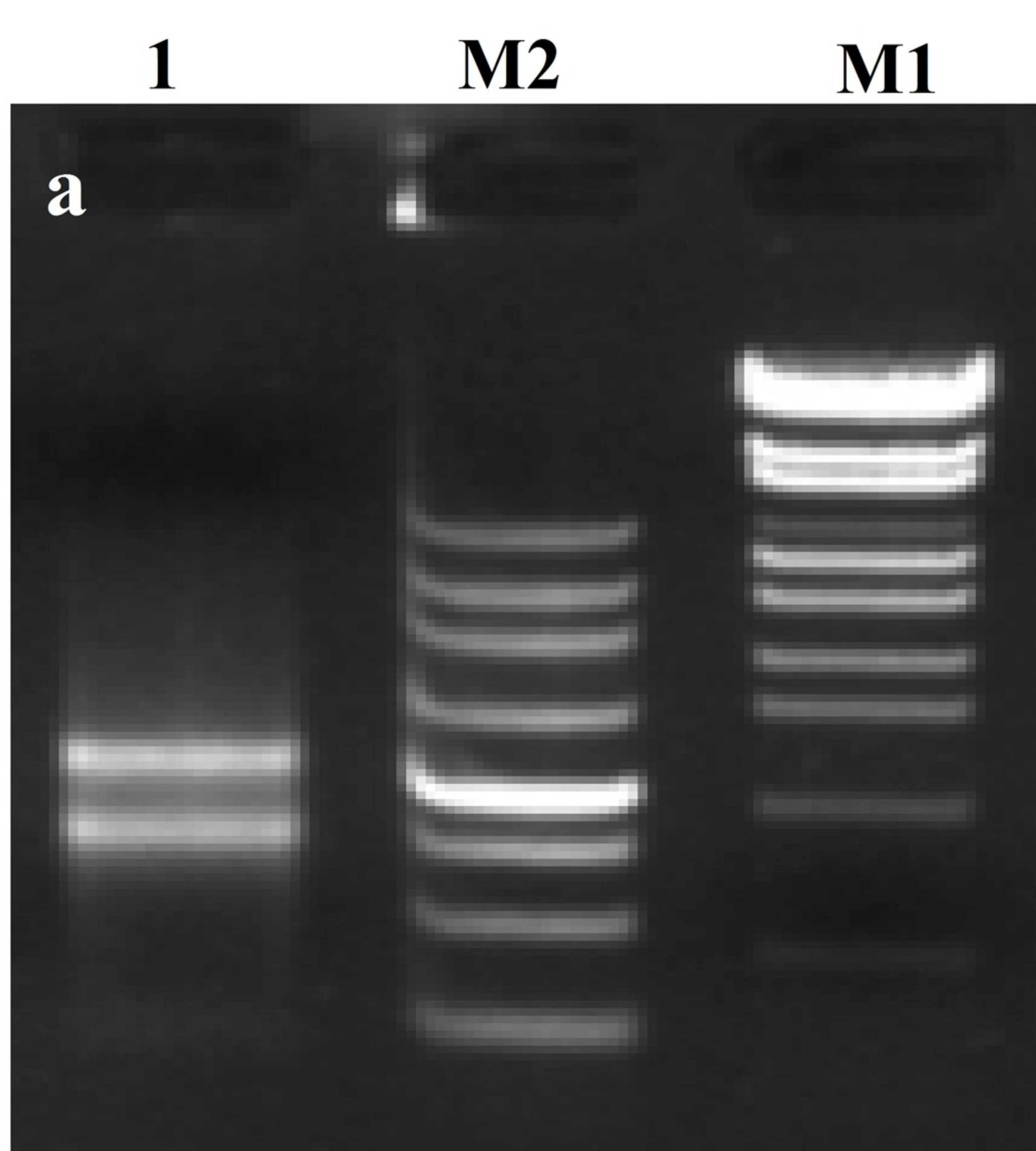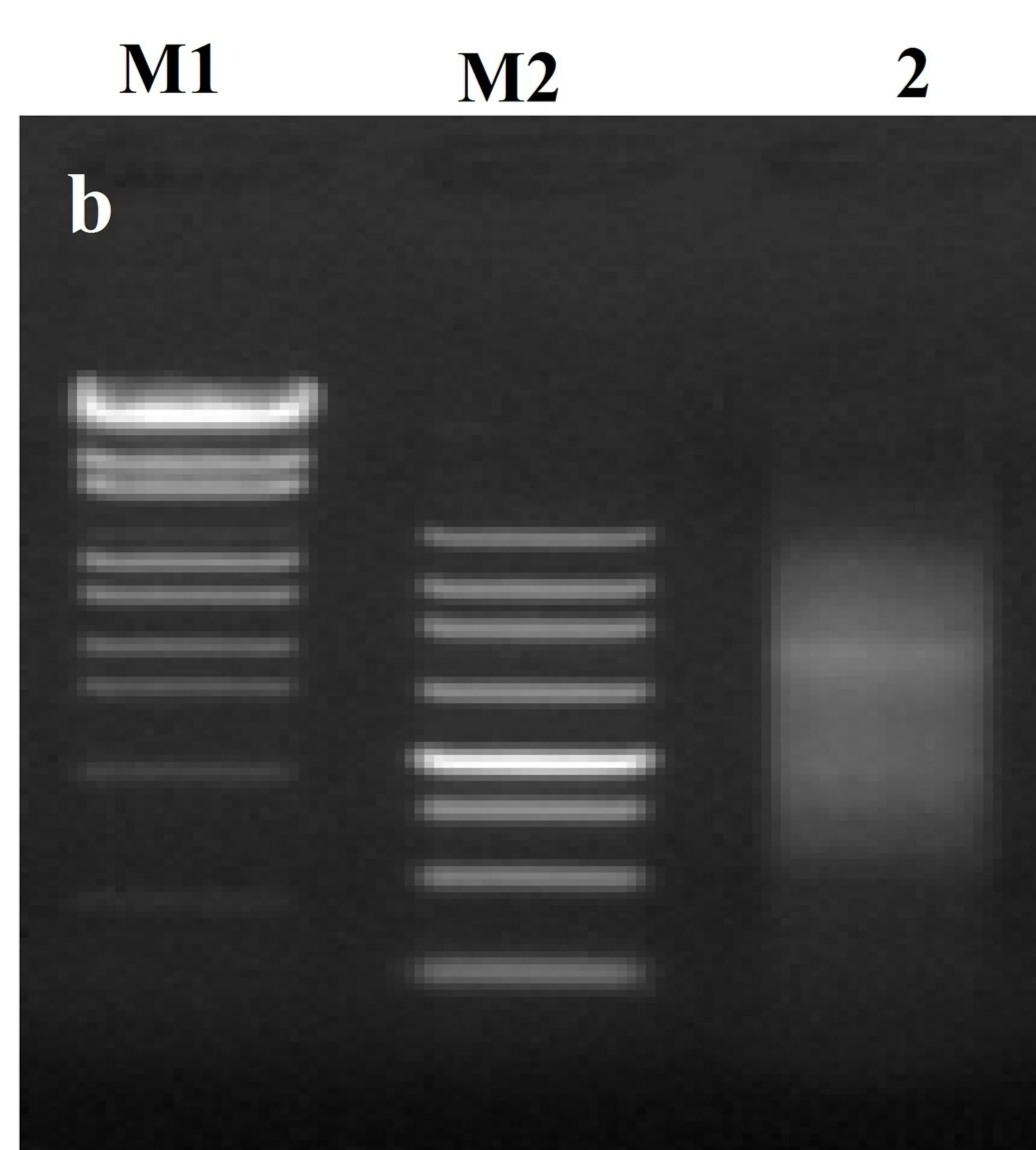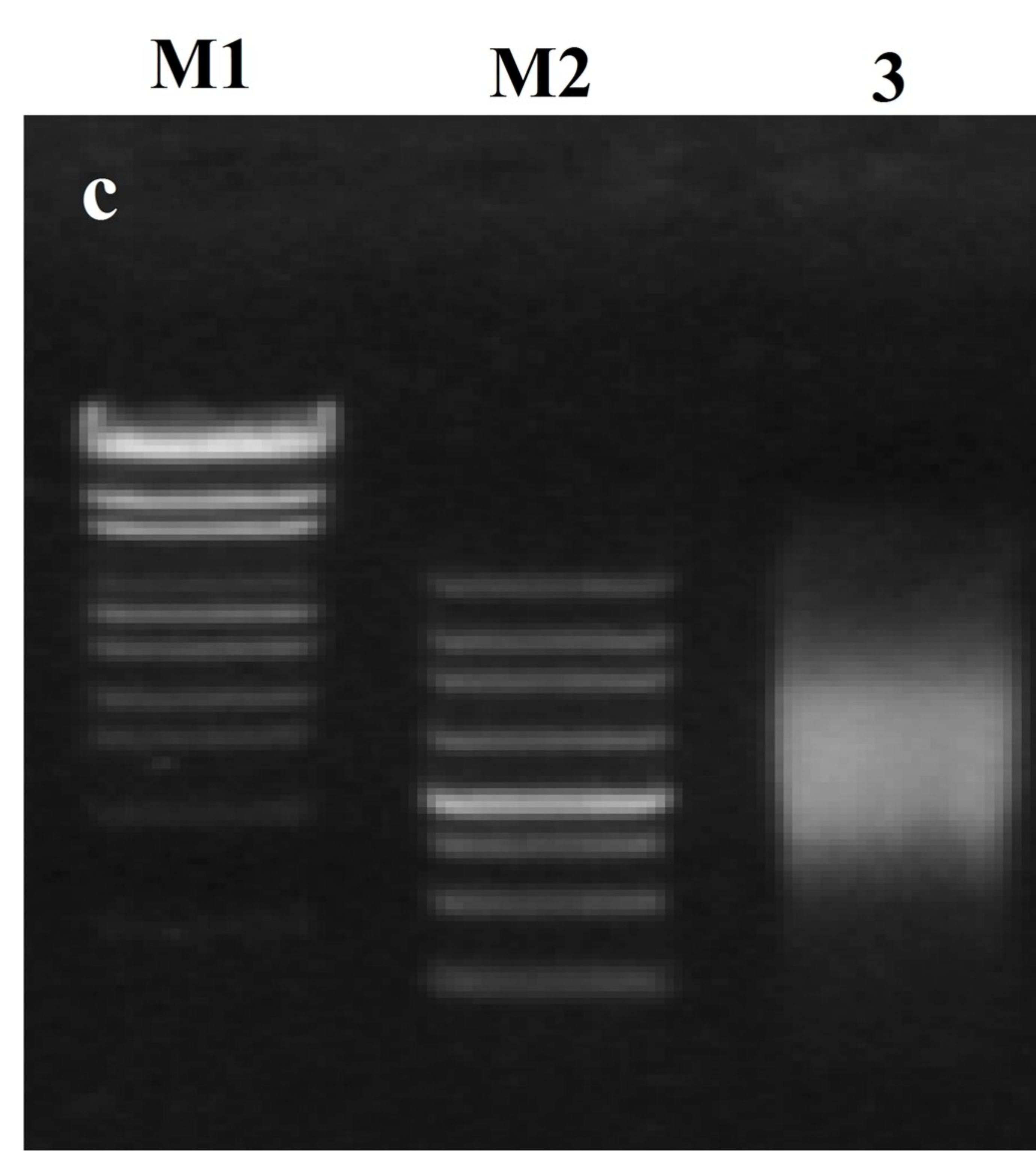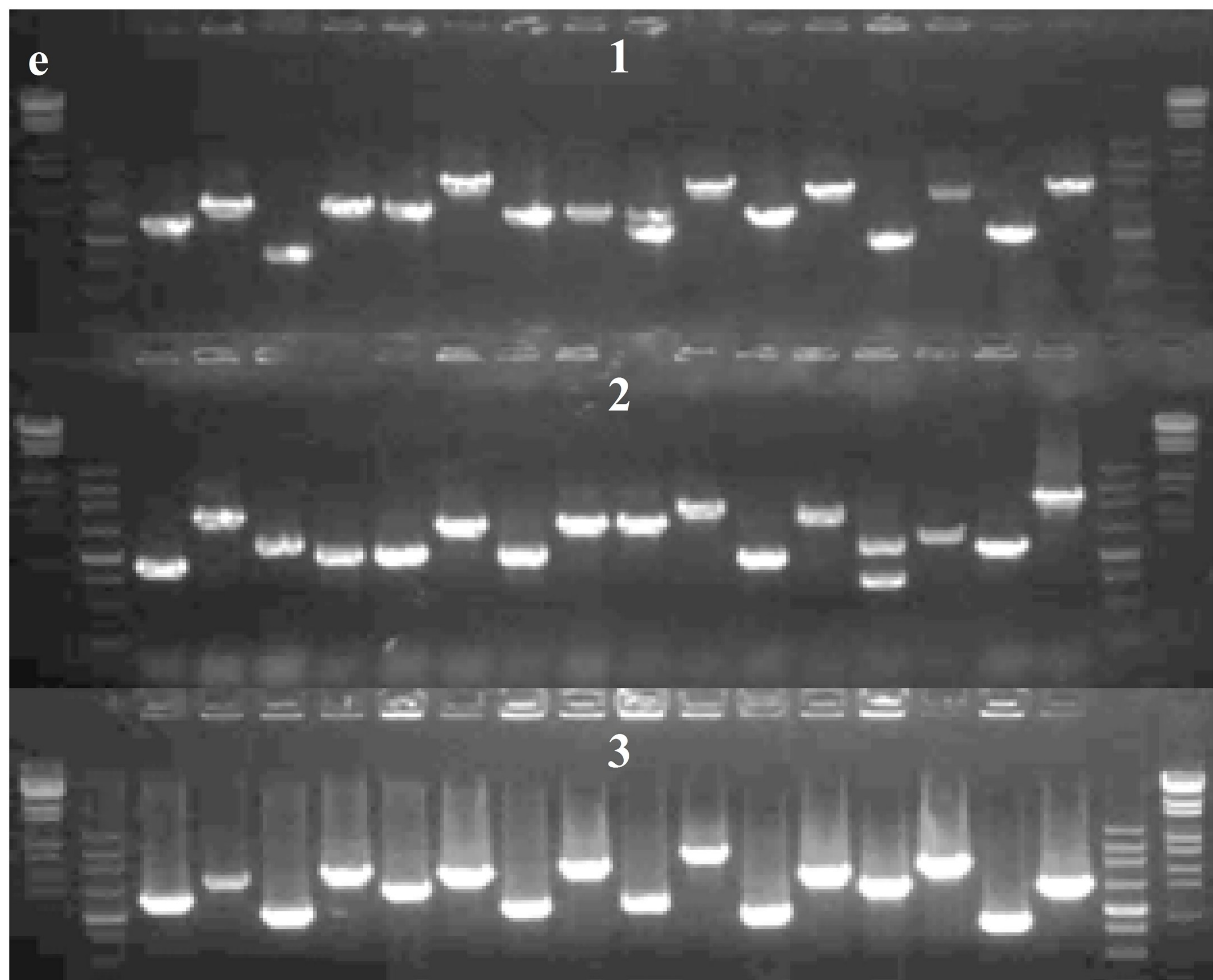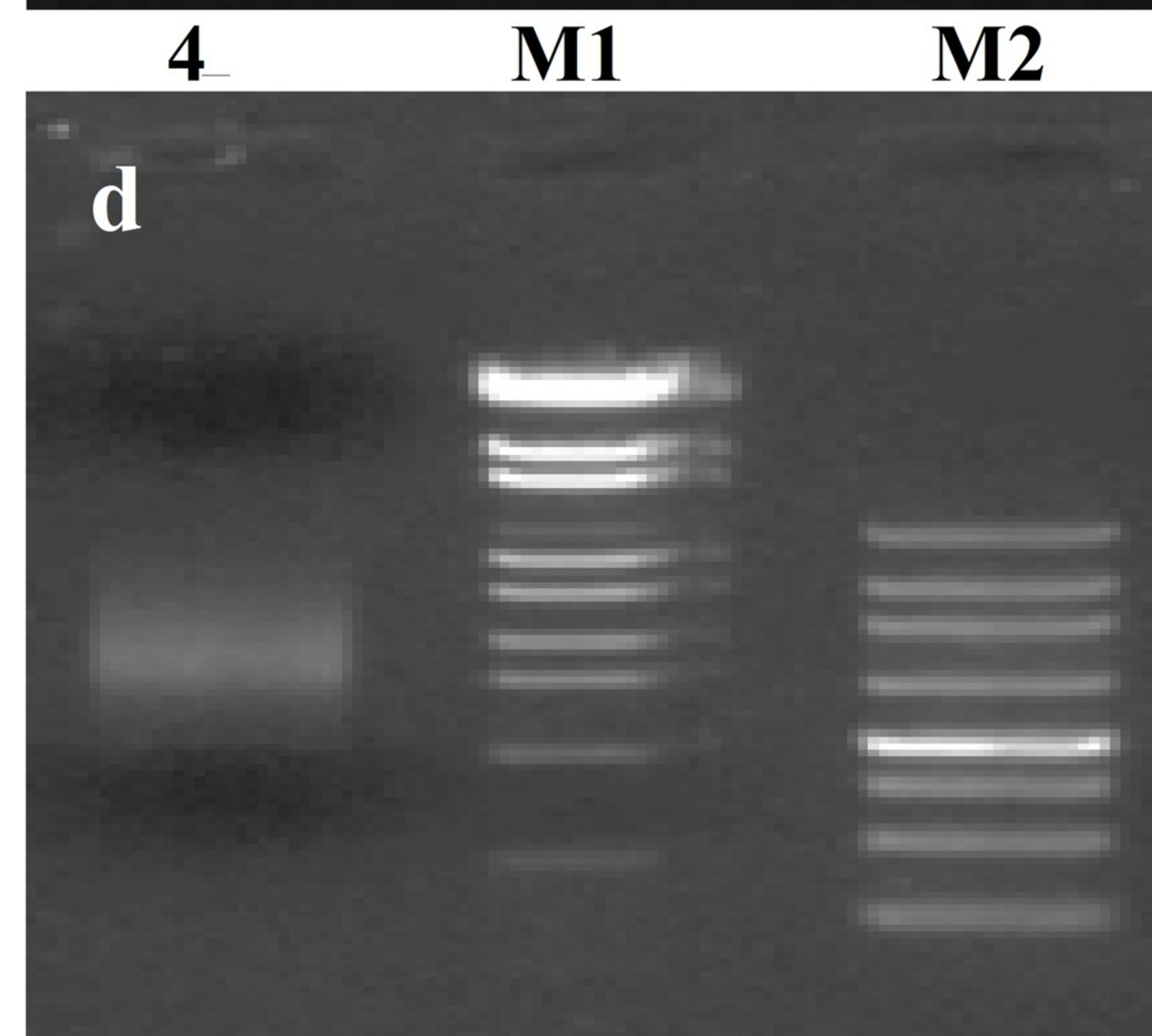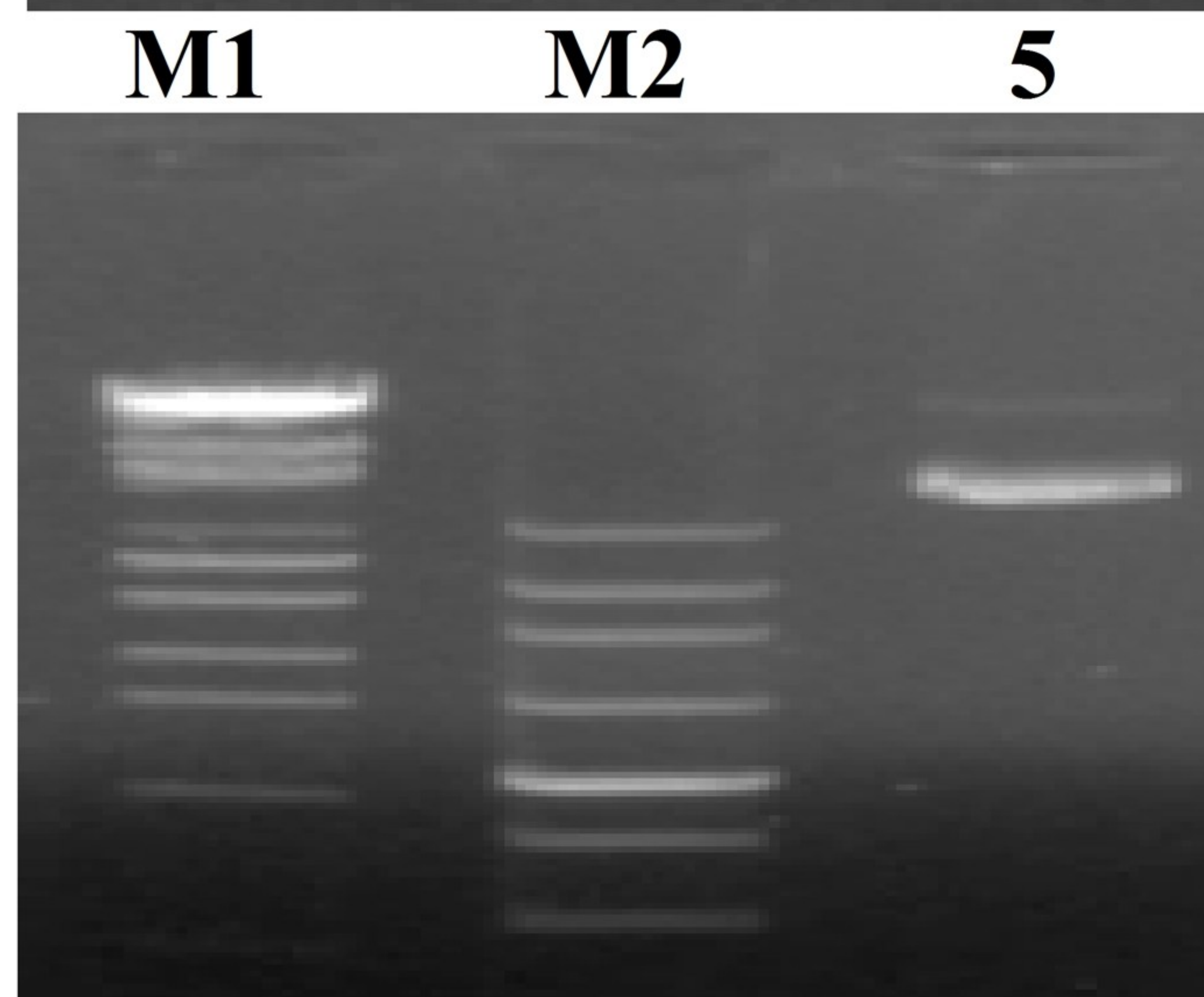

Supplement: Supplementary file 1 — Supplementary data to this article can be found online. [file FR-2021-0021-S1.zip › 10.48130_FR-2021-0021-Suppl-FigureS1.pdf]

**M**

**CLA354-WRKY31**

**M**

**CLA354-WRKY31**

**M1**

**M**

**CLA420-WRKY31**

**M**

**CLA420-WRKY31**

**a**

**b**

**c**

**d**

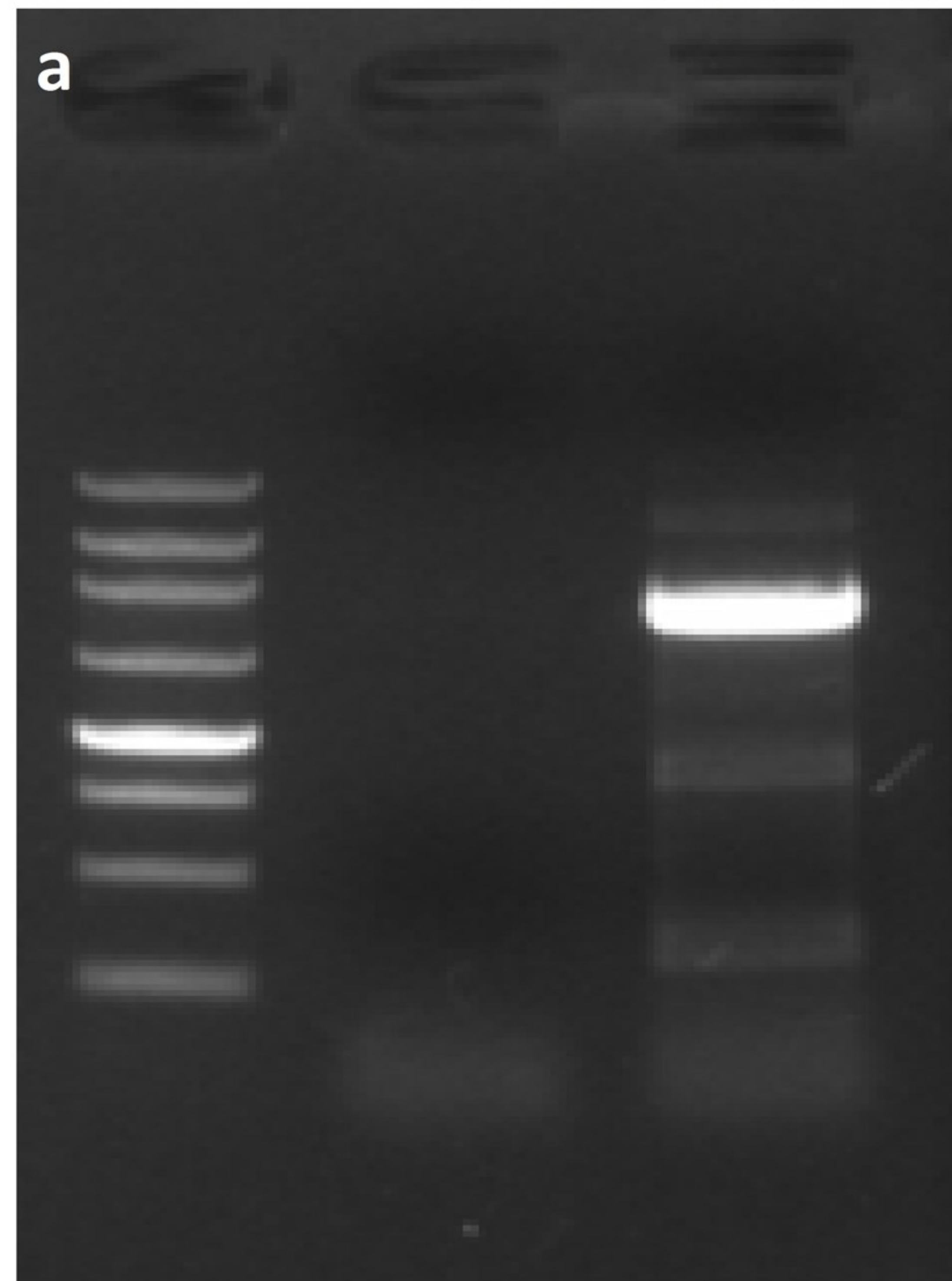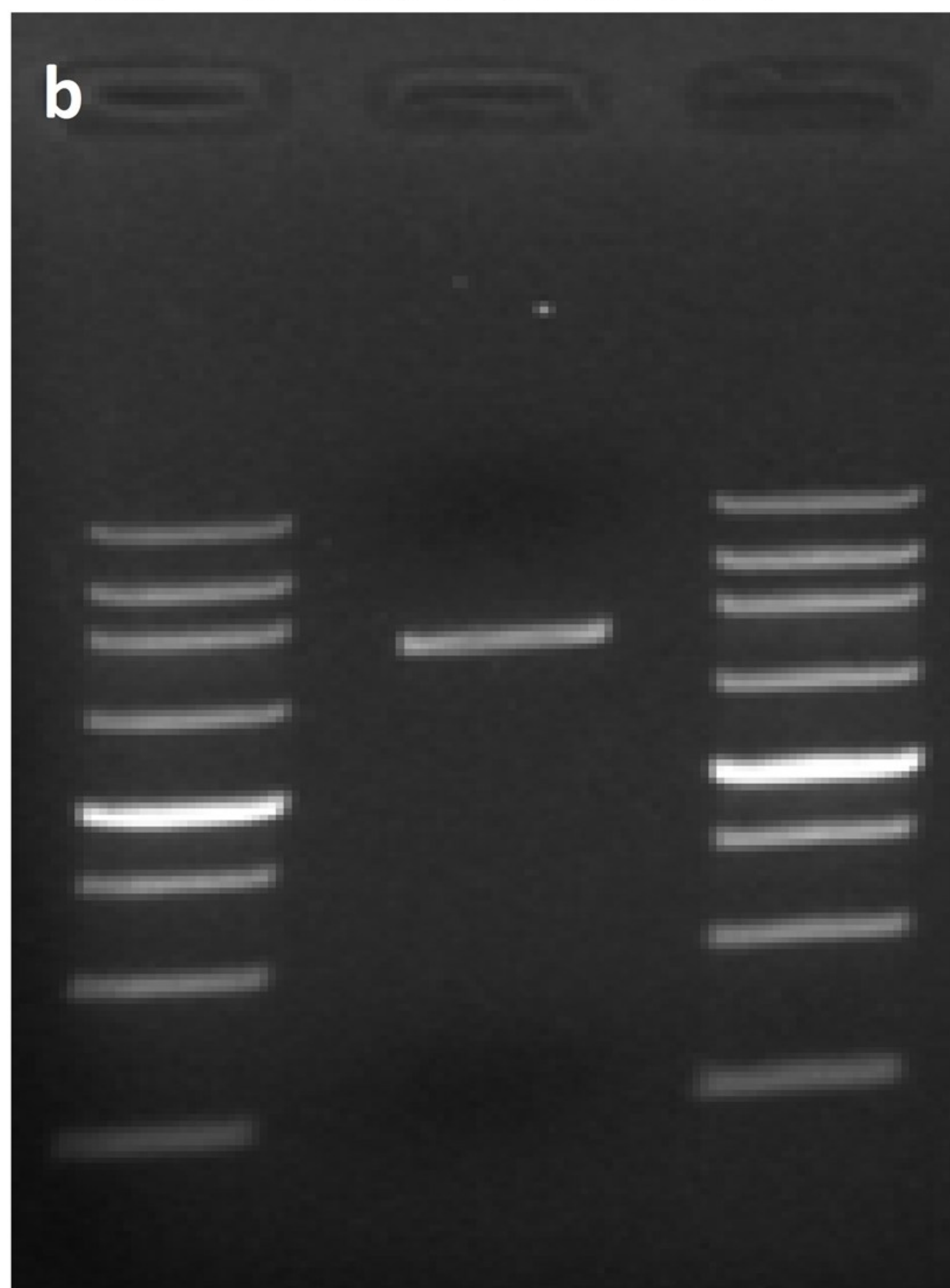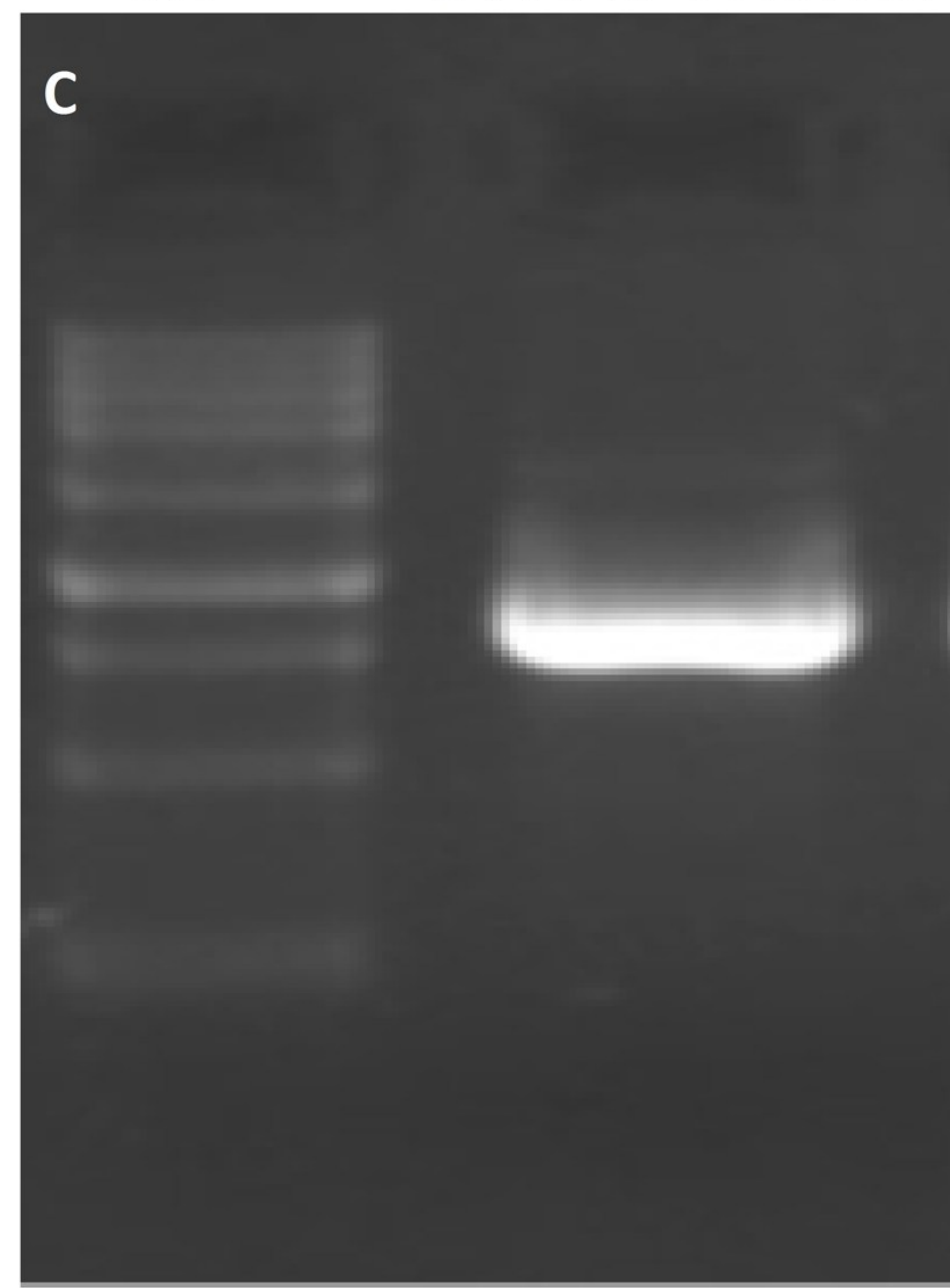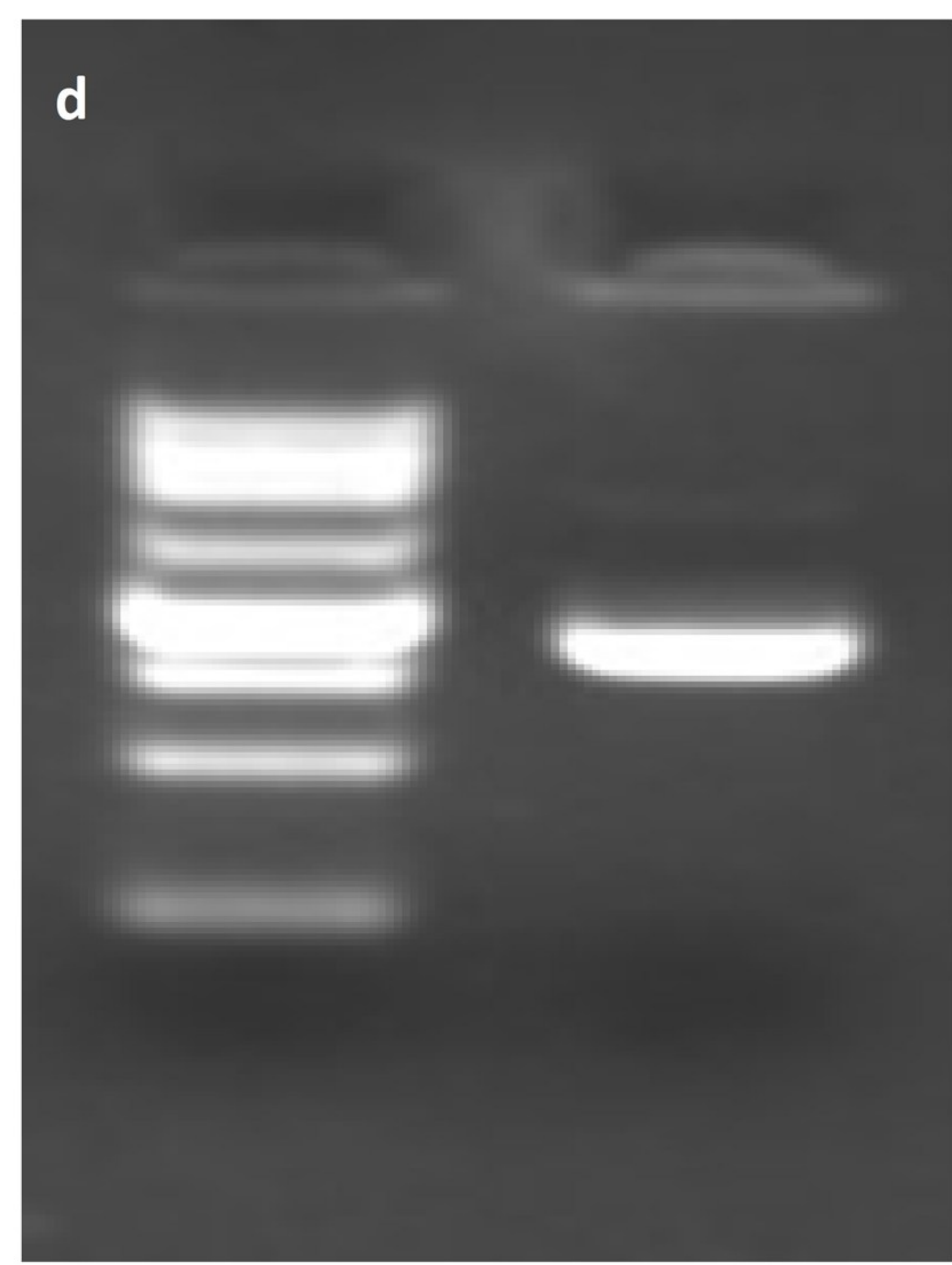

Supplement: Supplementary file 1 — Supplementary data to this article can be found online. [file FR-2021-0021-S1.zip › 10.48130_FR-2021-0021-Suppl-FigureS2.pdf]

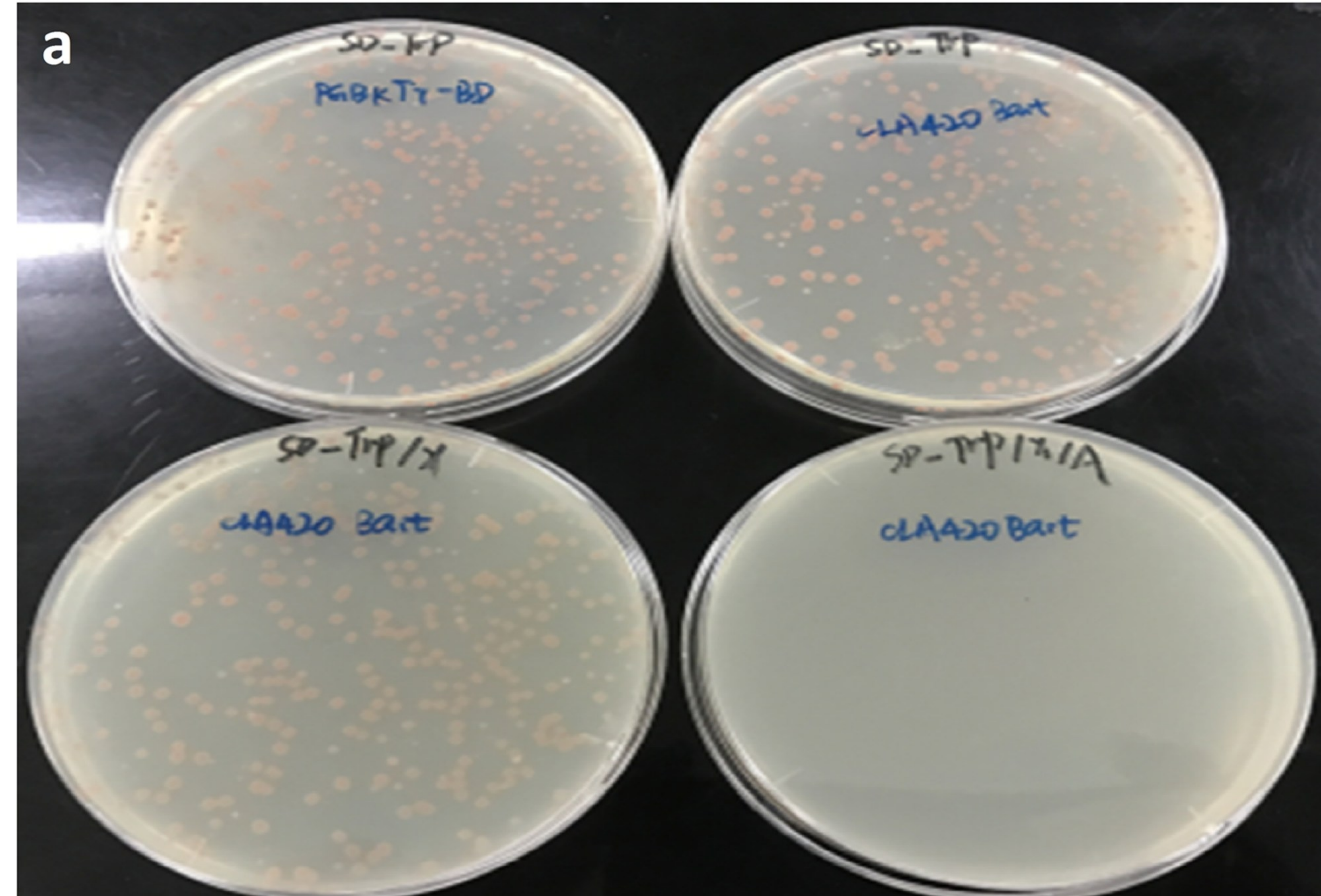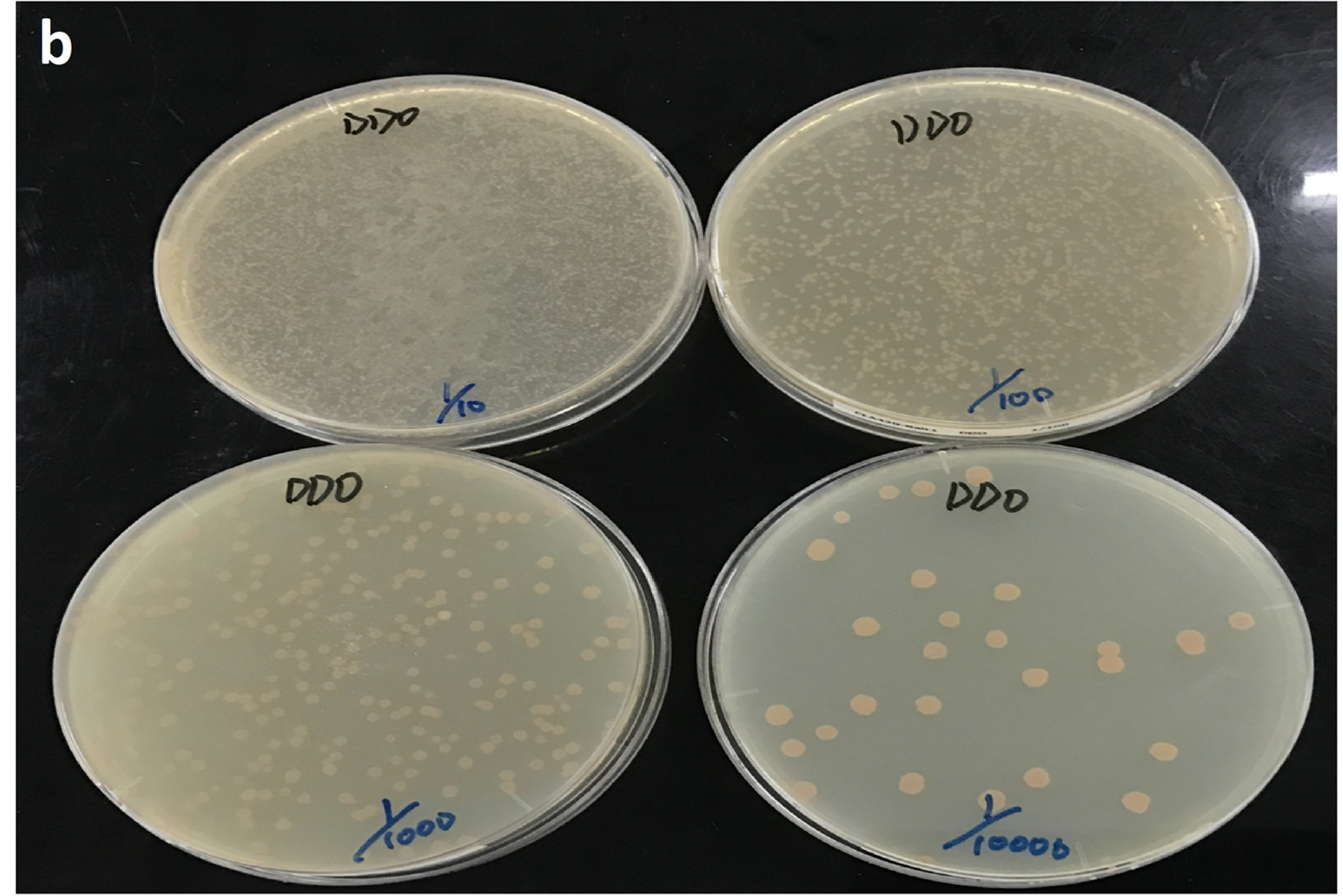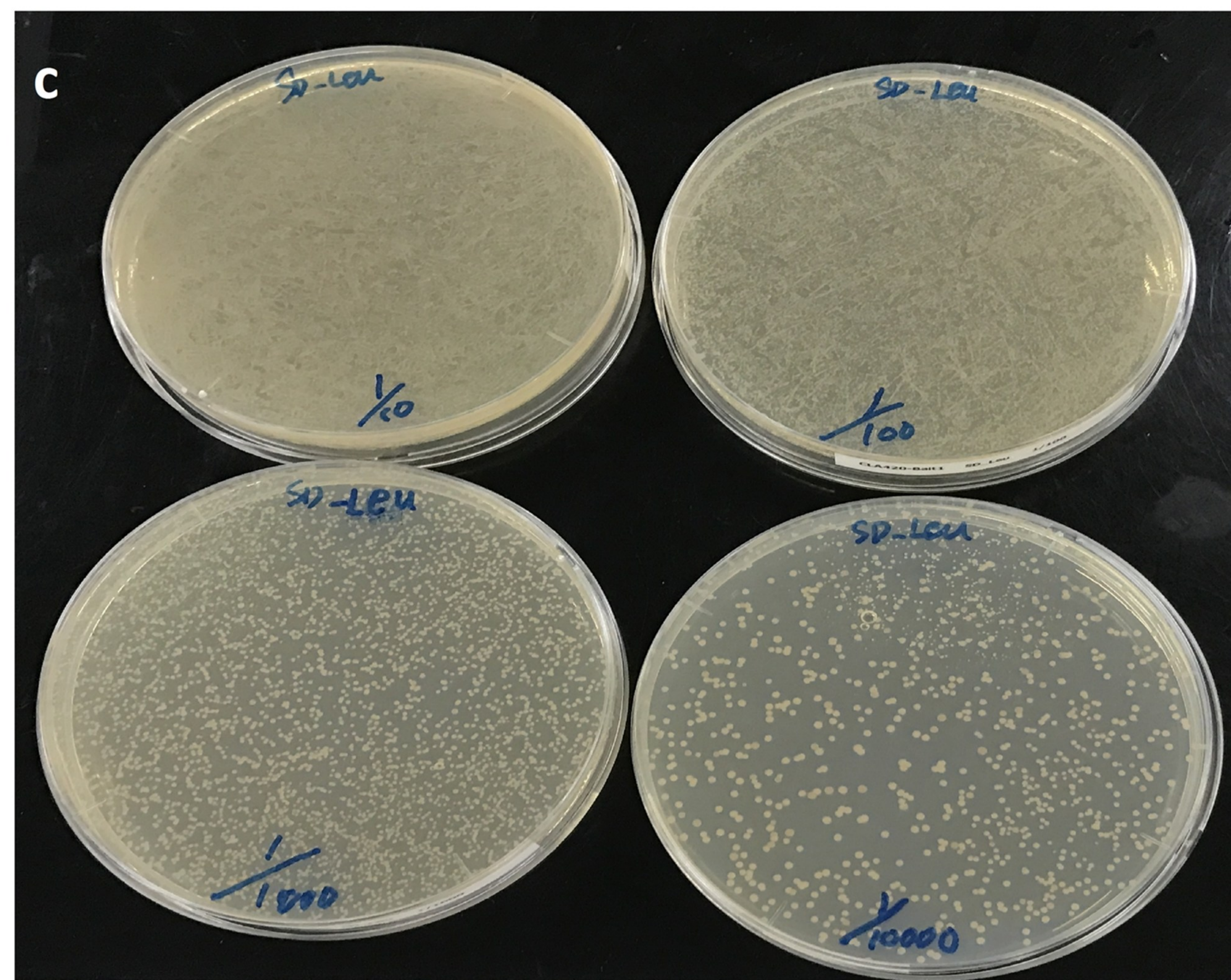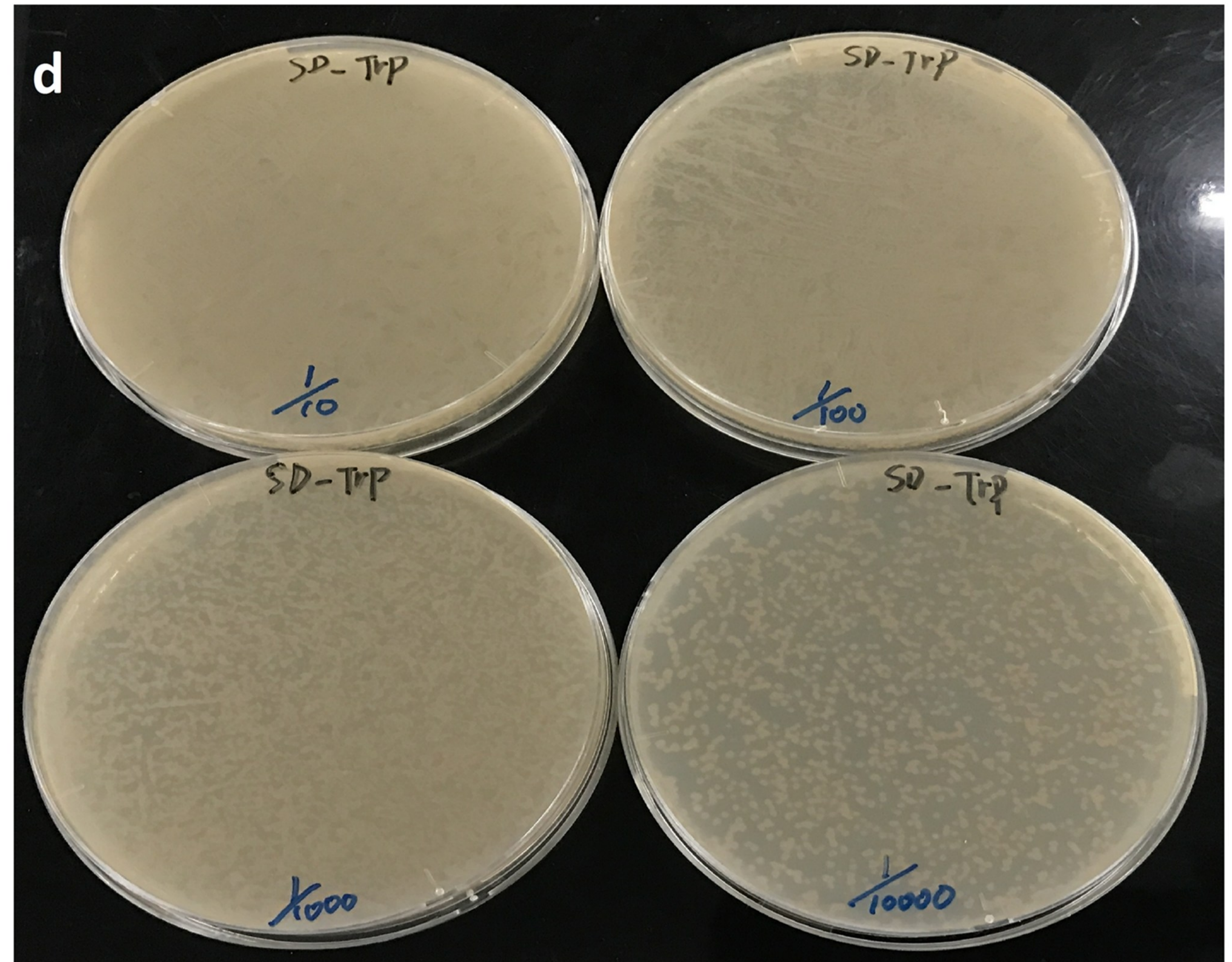

Supplement: Supplementary file 1 — Supplementary data to this article can be found online. [file FR-2021-0021-S1.zip › 10.48130_FR-2021-0021-Suppl-FigureS3.pdf]

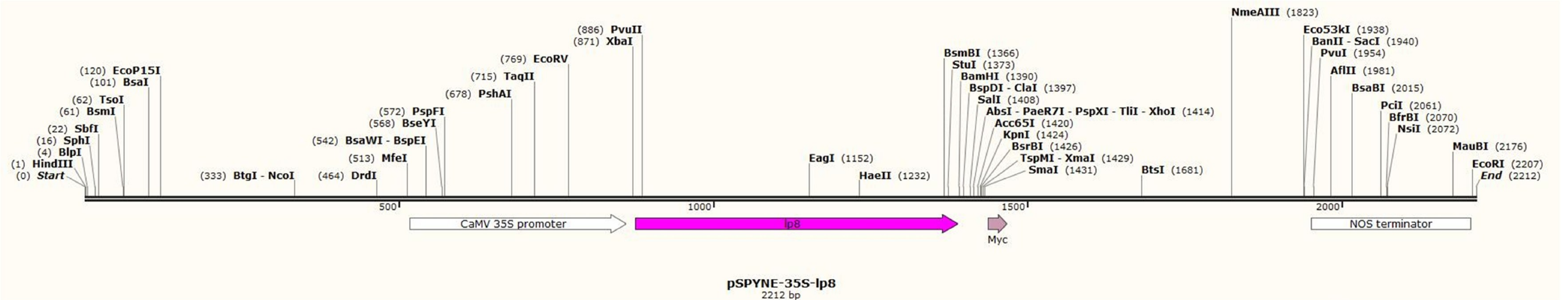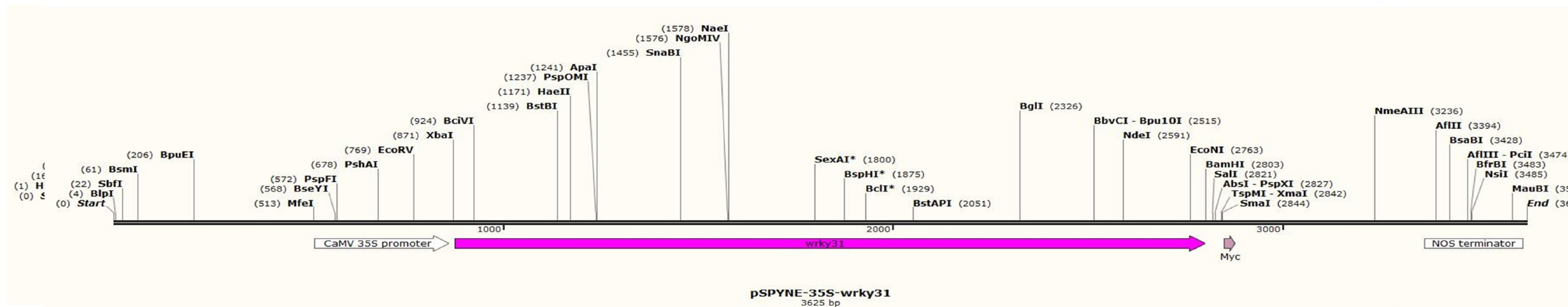

Supplement: Supplementary file 1 — Supplementary data to this article can be found online. [file FR-2021-0021-S1.zip › 10.48130_FR-2021-0021-Suppl-FigureS5.pdf]

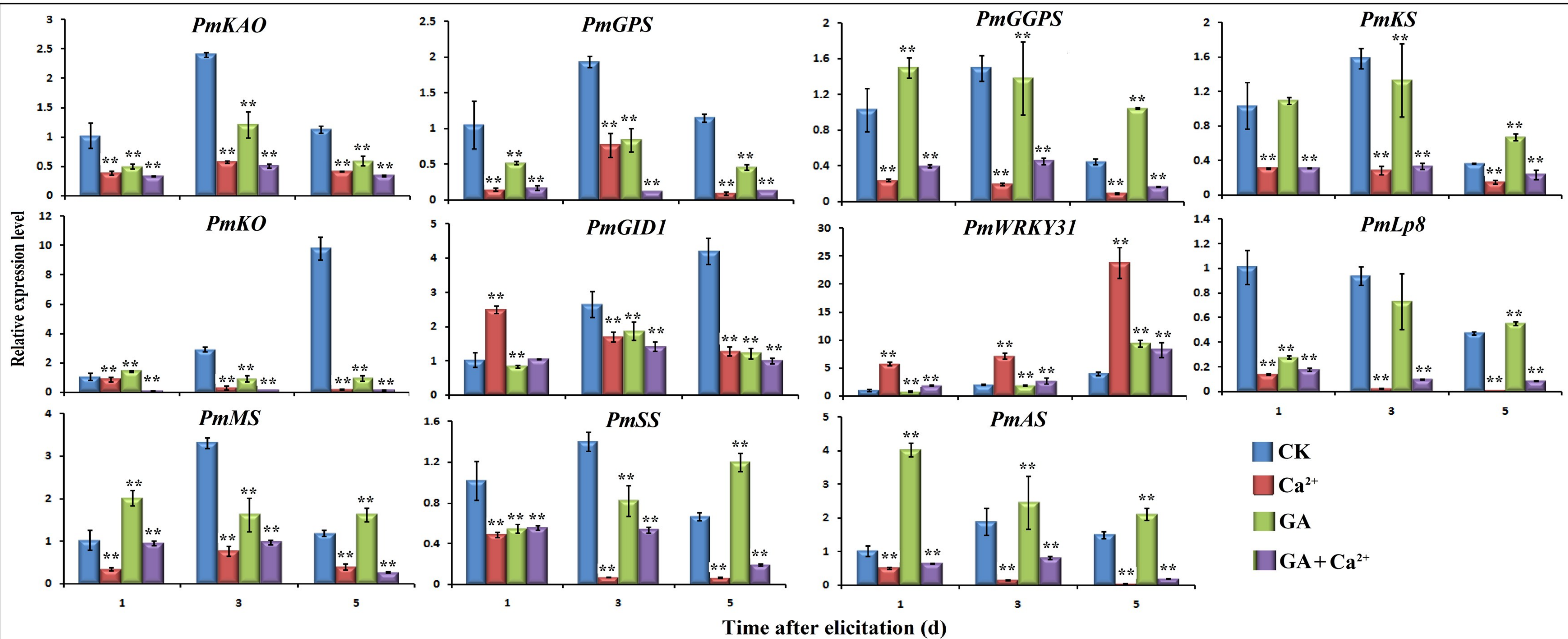

Supplement: Supplementary file 1 — Supplementary data to this article can be found online. [file FR-2021-0021-S1.zip › 10.48130_FR-2021-0021-Suppl-FigureS6.pdf]

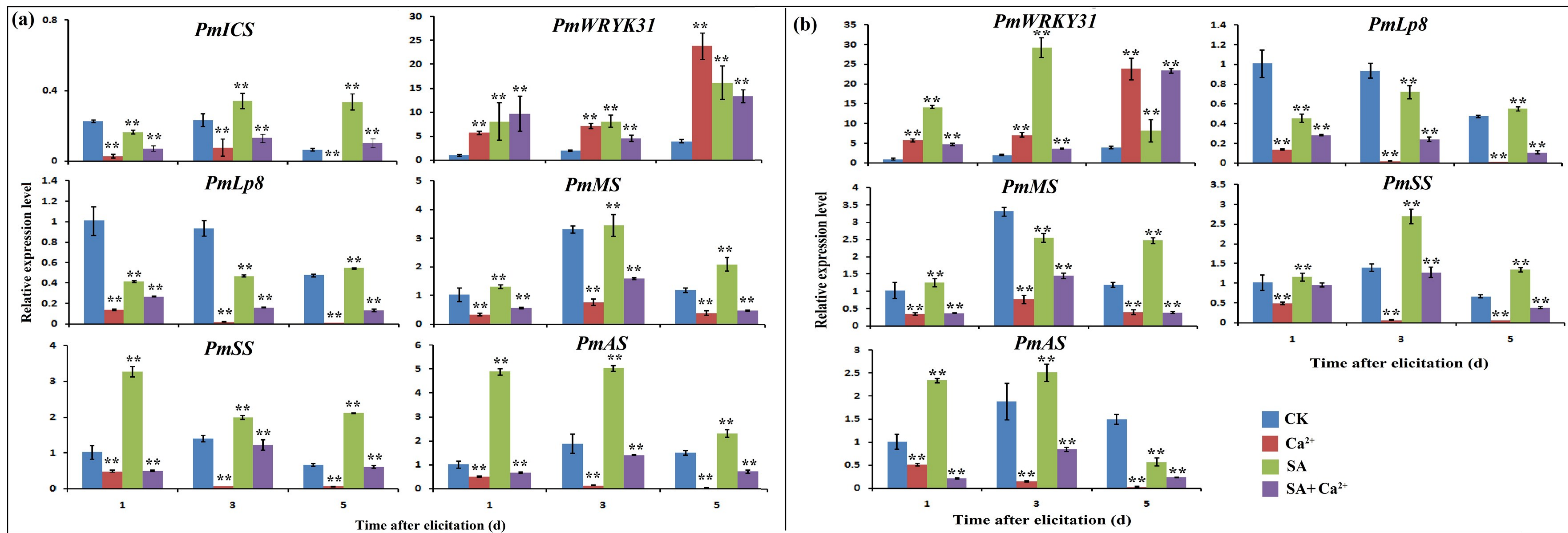

Supplement: Supplementary file 1 — Supplementary data to this article can be found online. [file FR-2021-0021-S1.zip › 10.48130_FR-2021-0021-Suppl-FigureS7.pdf]
